# Data Reduction Methodology for Dynamic Characteristic Extraction in Photoplethysmogram

**DOI:** 10.3390/s25196232

**Published:** 2025-10-08

**Authors:** Nina Sviridova, Sora Okazaki

**Affiliations:** 1 Department of Intelligent Systems, Tokyo City University, 1-28-1 Tamazutsumi, Setagaya-ku, Tokyo 158-8557, Japan; 2 International Research Center for Neurointelligence, The University of Tokyo, 7-3-1 Hongo Bunkyo-ku, Tokyo 113-0033, Japan

**Keywords:** photoplethysmography, nonlinear dynamics, nonlinear time series analysis, data reduction, computational efficiency, wearable devices

## Abstract

Photoplethysmogram (PPG) signals are increasingly utilized in wearable and mobile healthcare applications due to their non-invasive nature and ease of use in measuring physiological parameters, such as heart rate, blood pressure, and oxygen saturation. Recent advancements have highlighted green-light photoplethysmogram (gPPG) as offering superior signal quality and accuracy compared to traditional red-light photoplethysmogram (rPPG). Given the deterministic chaotic nature of PPG signals’ dynamics, nonlinear time series analysis has emerged as a powerful method for extracting health-related information not captured by conventional linear techniques. However, optimal data conditions, including appropriate sampling frequency and minimum required time series length for effective nonlinear analysis, remain insufficiently investigated. This study examines the impact of downsampling frequencies and reducing time series lengths on the accuracy of estimating dynamical characteristics from gPPG and rPPG signals. Results demonstrate that a sampling frequency of 200 Hz provides an optimal balance, maintaining robust correlations in dynamical indices while reducing computational load. Furthermore, analysis of varying time series lengths revealed that the dynamical properties stabilize sufficiently at around 170 s, achieving an error of less than 5%. A comparative analysis between gPPG and rPPG revealed no significant statistical differences, confirming their similar effectiveness in estimating dynamical properties under controlled conditions. These results enhance the reliability and applicability of PPG-based health monitoring technologies.

## 1. Introduction

In recent years, with the increasing use of wearable devices and mobile devices in healthcare, the number of devices measuring the photoplethysmogram (PPG) signal has been on the rise [1,2,3,4]. PPG is a biological signal used in clinical practice and health monitoring, representing the pulse waves. The signal acquisition mechanism involves measuring changes in blood vessel volume that happen when the heart pumps blood by shining light onto the skin and detecting fluctuations in the transmitted or reflected light [4,5]. The measurement is noninvasive and easy to perform. In the past, red and near-infrared light were used for PPG; however, over the last two decades, green light has been attracting attention due to its higher accuracy in detecting changes in blood flow, lower susceptibility to noise, and suitability for measurement at various locations [6,7,8,9,10]. Green light PPG (gPPG) has been shown to be equally promising for various applications as red light PPG (rPPG) [7,10,11].

PPG data can be analyzed to obtain relevant cardiovascular information, such as heart rate, blood pressure, oxygen saturation, and vascular stiffness index [4,7,12]. It has also been demonstrated that heart rate variability (HRV) derived from PPG can replace HRV measured by electrocardiography (ECG) [13,14]. Typical ECG measurements involve multiple electrodes and cables, which can cause discomfort to the subject and are not suitable for use in locations where electrical interference may occur. PPG, in contrast, is very effective as it causes minimal discomfort and is easy to use anywhere. Many studies have shown that PPG can also be used for the early detection of cardiovascular diseases [15,16,17,18] and mental health assessment [19,20,21,22], and thus can be utilized for professional health management. Additionally, PPG measurements are cost-effective and straightforward to operate, highlighting their suitability for everyday health monitoring. Moreover, advances in technology have enhanced the accuracy and miniaturization of PPG sensors, enabling the collection of more health data during daily activities and supporting self-care.

PPG, which is nowadays measured in various conditions, has been well investigated in terms of extracting heart rate variability (HRV). However, since the cardiovascular and respiration dynamics [23,24], especially the dynamics of PPG [11,25,26] have been shown to be deterministic chaos, nonlinear time series analysis has been applied in previous studies to extract information on health status directly from PPG dynamics. It has become clear that nonlinear dynamic features can be applied as health indicators [11,25,26,27]. Deterministic chaos is a phenomenon in which a system follows deterministic laws but exhibits unpredictable behavior, demonstrating a strong dependence on initial conditions and aperiodicity [28,29]. This type of information-rich and complex dynamics allows for the analysis of biological data, such as PPG signals, to identify potential health states that traditional linear models cannot capture.

Nonlinear time series analysis of PPG data is performed on data reconstructed into a delay coordinate system to capture the nonlinear dynamics of the system [29,30]. The analysis is then performed using various methods, such as recurrence plots, Lyapunov exponents, and fractal dimension analysis [29]. Recurrence plots (RPs) can be used to visualize and quantify nonlinear dynamic properties by performing recurrence quantification analysis (RQA) [31,32,33]. In PPG data, RQA was confirmed to provide robust and more sensitive results [34]. It has been used to improve the ability to estimate systolic and diastolic blood pressure [35], user authentication [36], stress assessment [22], discrimination between preterm and full-term newborns [34], and various other studies have validated it as a method for diagnosing health conditions (Table 1).

Many studies up to this point have shown the usefulness of applying nonlinear time series analysis to PPG data, but they have been used in analyses with various time series lengths, such as 2 s [37], 30 s [19], 100 s [22], 120 s [17], and 300 s [38]. In general, knowing the statistical properties of time series is a prerequisite for time series analysis, since infinitely long observation data can always reliably capture the statistical properties of the random variation of the target [29,39]. Therefore, a long time series length is also required in nonlinear time series analysis, and past studies have shown that RQA can usually be estimated more accurately with longer time series lengths [31,40]. However, in PPG, due to noise, movement artifacts, nonstationarity, and other effects, short time series are often used in the analysis [35,36].

To address the problem of the appropriate PPG time series length choice, a previous study [38] investigated the impact of rPPG time series length on the evaluation of its dynamical properties by RQA. In [38], it was demonstrated that the RQA index converged as the time series length increased. However, while the approach was validated using numerical models, the comparison was made with 120 s as the standard reference for time series lengths that varied up to 120 s, which ensured error convergence to zero but was insufficient to determine a lower error limit. As seen in Table 1, considering the time series length used in various applied studies, the reference time series length in [41] was short and insufficient as an indicator for accurate estimation. Moreover, as the reference time series length in the previous study was short, an important problem of nonstationarity in PPG data was left undiscussed. Also, from the perspective of the PPG signal itself, the characteristic differences between various wavelengths of light sources, their effects on dynamic status evaluation, and the optimal time series length and sampling frequency for measurement and analysis remain open for discussion. In this study, to facilitate more advanced analysis using PPG with reduced data, we utilize gPPG and rPPG to investigate the impact of PPG data time series length and compare them across different sampling frequencies using RQA. Using shorter time series can significantly optimize the computational load when performing nonlinear time series analysis, such as RQA. Additionally, optimizing the sampling frequency may help improve energy consumption efficiency in wearables, which is an important issue that needs attention in battery-powered devices. Moreover, complex dynamics preserving optimization of data measurement requirements, especially in sampling rate, may further contribute to the rapidly developing area of battery-free body implants, for which achieving energy consumption efficiency is one of the essential problems [42].

**Table 1:** Overview of PPG time series length used in previous studies.

Topic of Case Study	Time Series Length Used
Distinction between normal blood pressure and hypertension [37]	2.1 s
Estimation model of systolic and diastolic blood pressure [35]	2~3 s
Subject authentication method [36]	7 s
Love at first sight impulse detection [43]	10 s
Analgesia depth during anesthesia [44]	10 s
Blood Pressure Estimation [15]	20 s
Mental health assessment [19]	30 s
Correlation with fear/anxiety [45]	30 s
Automatic sleep staging [46]	30 s
Blood sugar estimation [47]	60 s
Early detection of cardiovascular disease [16]	60 s
Automatic Emotion Recognition [48]	60 s
Effects of Mental Stress [22]	100 s
Fatigue Detection [49]	120 s
Estimation of cardiovascular age [17]	120 s
Automatic detection of hypertension [18]	120 s
PPG time series length criteria [41]	120 s
Early detection of depression [21]	180 s
Estimation of blood glucose level [50]	180 s
Effects of changes in gestational age [51]	180 s
Effects of mental illness [20]	180 s
The rPPG dynamics investigation [25]	300 s
The rPPG and gPPG dynamics investigation [11]	300 s
Estimation of blood pressure [38]	300 s
Early hypertension detection [52]	300 s
Effects of tractor noise on the cardiovascular system [53]	300 s
Variation of fatigue during driving [54]	300 s
Comparison between surgical patients and healthy subjects [55]	300 s
Detection of sleep apnea syndrome [56]	300 s

Therefore, the purpose of this study is to investigate the impact of data reduction methods on extracting dynamic characteristics from gPPG and rPPG data in two main aspects. The first is data down-sampling. In this study, comparisons are made between the common data sampling frequencies used in PPG devices: 400 Hz, 200 Hz, and 100 Hz. Second, data with varying time series length: compared to the previous study [41], a longer time series length is used as a standard for comparison. Thus, the reference value in this study is 300 s, which is used in many advanced PPG-related studies, as shown in Table 1. At the same time, this duration provides signal stationarity, as will be thoroughly discussed in Section 2.4.

This study investigates the effects of these two points using gPPG and rPPG to clarify their differences in data requirements. It also aims to propose minimal suitable frequency and time series length settings for estimating PPG dynamic features through nonlinear time series analysis methods.

## 2. Data

### 2.1. Photoplethysmogram Signal

**Figure 1:**
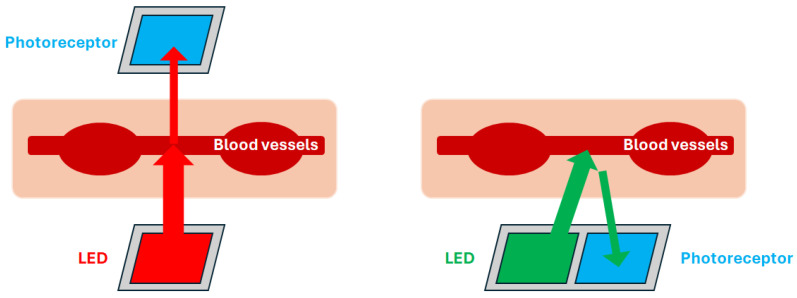
Illustration of PPG recording mechanism (**left**: transmission type, **right**: reflection type).

Traditionally, photoplethysmography measures pulse waves by irradiating a light source onto the skin of a fingertip, earlobe, arm, etc., causing the light to be partially absorbed by the body’s tissues and detecting fluctuations in transmitted or reflected light. The PPG waveform formation mechanism is based on the fact that with each heartbeat, the blood volume in the vessels varies, affecting the rate of light absorption. The PPG recorded with transmitted light is called the transmission type and is measured using a fingertip or earlobe, through which light can pass. On the other hand, those that use reflected light are called reflection type and can be measured not only on the fingertips but also on the arms and other parts of the body. Near-infrared light (900 nm), red light (660 nm), and green light (530 nm) are commonly used as the light source [11]. When infrared or red light is used, its application is limited due to the influence of sunlight on infrared light. However, green light, which is less affected by external disturbances, has a high light absorption rate and is therefore suitable for outdoor use [57]. An illustration of each type of measurement method is shown in Figure 1.

The PPGs used in this study are two commercially available setups: the reflection-type gPPG and the transmission-type rPPG, which is similar to the rPPG signals used in [41]. The recording was performed using a green light (530 nm) pulse wave sensor (Arduino [58]) with a recommended sampling frequency of 500.0 Hz for gPPG, and a red (660 nm) and near-infrared (900 nm) pulse wave sensor with a factory-set sampling frequency of 409.6 Hz (Tokyo Devices, Inc., Tokyo, Japan) for rPPG.

### 2.2. Data Collection Experiment

**Figure 2:**
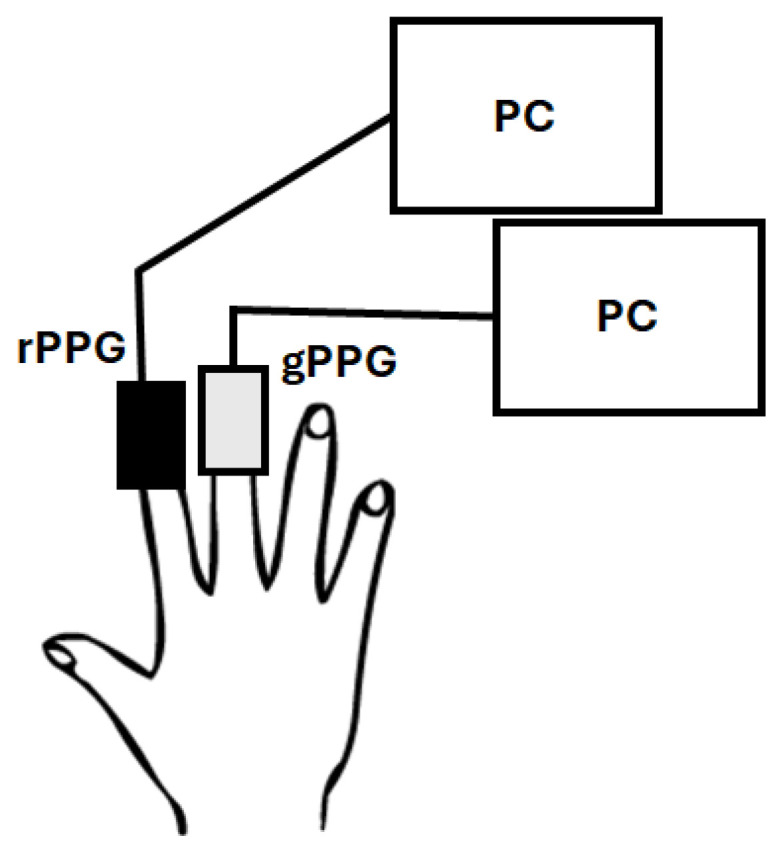
Experimental setup during data recording.

The subjects of the experiment were 18 healthy male and female students from Tokyo City University, all in their 20 s, with no history of heart disease. After explaining the purpose of the study, the experimental method, and the associated risks, all subjects were asked to sign a consent form and provide their consent to participate in the experiment. The experiment was conducted in a quiet room maintained at room temperature (24 ± 1 °C). Before the start of the experiment, the subjects’ blood pressure and heart rate were measured using a digital blood pressure monitor (Omron HCR-7106, Omron Healthcare Co., Ltd., Kyoto, Japan) to confirm that they were within normal limits (systolic blood pressure 90–129 mmHg and diastolic blood pressure less or equal than 80 mmHg [59]). After a short rest period, the resting state of each subject was measured for 10 min. If the measurements were not successful, they were repeated at a later time. The subjects were instructed to remain in a sitting position and to move as little as possible. As shown in Figure 2, rPPG was attached to the index finger of the right hand and gPPG to the middle finger. According to a previous study [5], differences in measurement position between these two fingers do not cause a significant difference in the PPG signal when recorded from healthy subjects.

### 2.3. Data Preprocessing

In this study, all data preprocessing and analysis were conducted using Python 3.12 programs on a Mac Studio (Apple M2 Ultra, 64 GB, Cupertino, CA, USA).

In this study, the sampling frequencies of the acquired PPG data are 500 Hz for gPPG and 409.6 Hz for rPPG. The PPG signals are measured at various sampling frequencies, with 100–1000 Hz used in the analysis [8,14,18,22,36]. Past studies have shown that 5 Hz is sufficient to calculate the average heart rate from PPG [60]. However, for analysis using the entire pulse waveform, the necessary sampling frequency is unclear, and different values are used as described above.

The PPG amplitude spectrum obtained through the Fourier transform, which shows the strength of each frequency component of the signal, is displayed in Figure S1. According to the sampling theorem, a sampling frequency of at least twice the highest frequency of the signal is necessary. Specifically, a sampling frequency of at least 10 times is sufficient to accurately display and record waveforms [29].

For this reason, the frequencies compared in this study are 400 Hz to match the rPPG device with the highest sampling frequency, followed by 2× and 4× downsampling, i.e., 200 Hz and 100 Hz.

All PPG signals underwent preprocessing that involved first removing trends, then filtering with a fourth-order Butterworth filter [61,62] to eliminate noise and extract the pulse wave shape, as used in previous studies [8,41,48,62]. Figure S2 illustrates the frequency response of the one to five orders Butterworth filter. Figures S3 and S4 display comparisons of raw and filtered PPG data for gPPG and rPPG, respectively.

### 2.4. Data Selection

#### 2.4.1. Quality of PPG Data

When handling PPG, noise can easily be generated, which may negatively impact measurement accuracy and lead to incorrect results. Additionally, it is important to ensure that the quality of the PPG data obtained is maintained throughout the 10-min measurement period in this study. Therefore, we evaluated the measured PPG data using skewness, which has been shown in previous studies to be an effective quality index [63,64]. Skewness ($S_{SQI}$) measures the symmetry or asymmetry of a probability distribution and is defined by Equation (1).

(1) $$S_{SQI}=\frac{1}{N}\sum_{i=1}^{N}{\left[\frac{x_{i}-{\hat{u}}_{x}}{\sigma }\right]}^{3},$$

where *N* is the number of data points in the signal, ${\hat{u}}_{x}$ is the mean of the data, and σ is the standard deviation of the signal. This measure detects abnormal changes in noisy PPG signals [63], and a time series length of 5 s is sufficient for its calculation [64].

**Figure 3:**
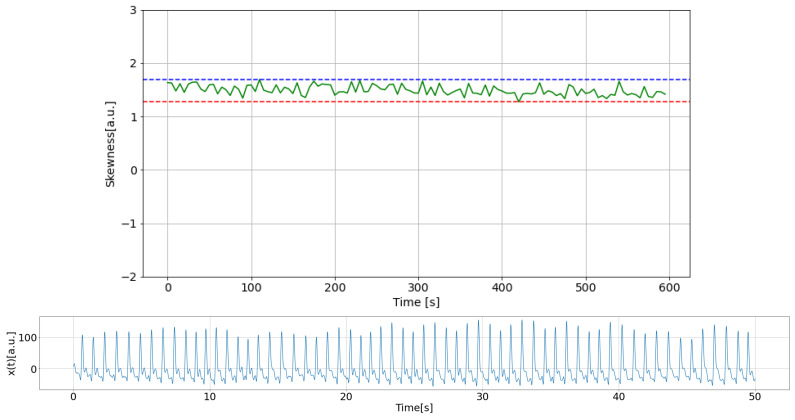
An example of a normally measured PPG time series: (**top**) Skewness with dashed lines indicating its high (blue) and low (red) limits; (**bottom**) part of PPG data.

**Figure 4:**
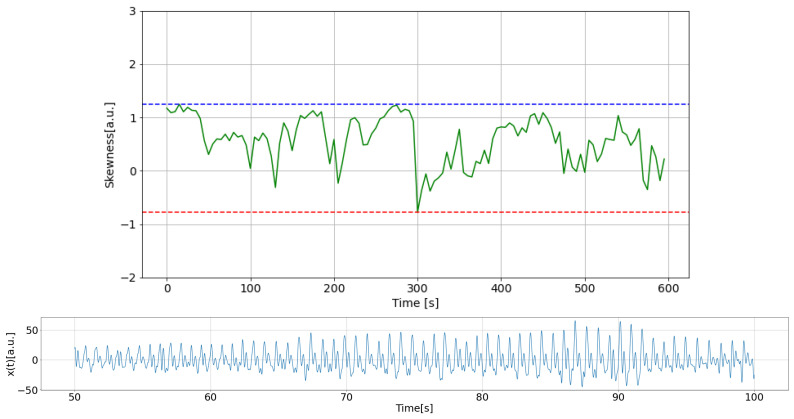
An example of PPG time series measurement failure: (**top**) Skewness with dashed lines indicating its high (blue) and low (red) limits; (**bottom**) part of PPG data.

In this study, comparisons were made over 600 s of data, shifting them in a 5-s window, and data showing abnormal changes were considered measurement failures. Figure 3 and Figure 4 show an example of a plot of the changes along with the corresponding PPG time series. The data sample shown in Figure 3 was identified as normal, while Figure 4 presents an example of a measurement failure.

#### 2.4.2. Estimation of Stationarity Through Heart Rate

Another issue that arises when analyzing long PPG time series is the potential nonstationarity, such as changes in the subject’s state due to mental load during measurement, which raises the question of whether the PPG data obtained reflect a single state or not. To address this issue, we first perform HRV analysis. Typically, when a person is under mental stress, the autonomic nervous system is affected [65]. HRV analysis is the most widely used and relatively efficient method for analyzing these changes. The waveform of PPG data consists of several peaks, and similar to electrocardiograms, the highest peak is called the R wave, and the interval between the R wave and the next R wave is called the RR interval (RRI). Figure S5 shows a typical PPG waveform, and Figure S6 shows the PPG waveform with the largest peaks plotted in red. The RRI is calculated by measuring the interval between these red peaks shown in Figure S6.

The RRI data can be used to calculate the heart rate (HR) using Equation (2).

(2) $$HR(bpm)=\frac{60}{RRI\left[s\right]}$$

**Figure 5:**
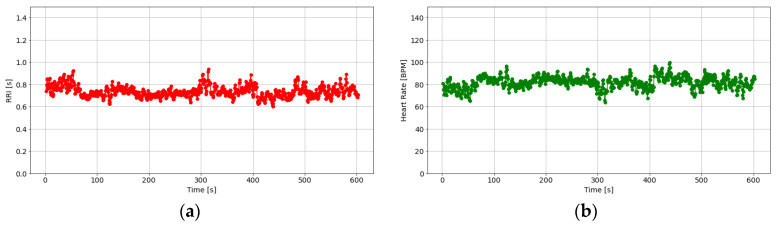
An example of heart rate variability estimation: (**a**) RRI; (**b**) heart rate.

An example of the RRI and HRs calculated from PPG data is shown in Figure 5. In general, the heart rate of a healthy adult ranges from approximately 60 to 100 beats per minute (bpm) [66], although there are individual differences.

#### 2.4.3. HRV Analysis

HRV analysis was performed using the extracted RRI data to determine if the obtained PPG data were stationary during data collection. HRV analysis includes both time-domain and frequency-domain indices [65]. In this study, analysis is conducted using these indices.

Generally, analysis methods using frequency-domain indices are difficult to manage because they cannot be applied to short time periods and require detailed parameter settings, as a measurement of five minutes or longer is standard [65]. In contrast, analysis methods using time-domain indicators need relatively shorter measurement times and can be performed with simple calculations [65]. Therefore, the subject’s state is assessed by calculating the frequency-domain index over a 5-min window and the time-domain index over a window with an RRI of 120 points.

First, LF/HF is computed from the power spectrum density as a frequency domain index. The power spectrum density can be calculated using either the fast Fourier transform or the maximum entropy method, from which the low-frequency component (LF) from 0.04 to 0.15 Hz and the high-frequency component (HF) from 0.15 to 0.4 Hz are derived. The LF/HF ratio is then used as an index. In previous studies, LF/HF values of 0 to 2 are considered good, 2 to 5 are seen as cautionary, and 5 or more are classified as very cautionary for assessing fatigue levels [67,68]. Evaluation is based on fluctuations in LF/HF, although individual differences may occur, such as in people with autonomic nervous system disorders.

Two examples of LF/HF for normal and unstable HRV data are shown in Figures S7 and S8.

Next, *sd2*/*sd1* was obtained from the Poincaré plot as a time domain index. The Poincaré plot, also called the Lorenz plot, is constructed with the nth RRI on the horizontal axis and the n + 1st RRI on the vertical axis. Figure S9 shows an example of a Poincaré plot. This is an example where the state is determined to have changed since 300 s. The standard deviation on the *y* = *−x* axis is *sd1*, the standard deviation on the *y* = *x* axis is *sd2*, and *sd2*/*sd1* is used as a time domain index. This value has a high correlation with LF/HF and can be treated as an index for stress analysis [65]. Similarly to LF/HF, evaluation is made based on the variation of this value. As an example, the standard deviations (*sd2*/*sd1*) obtained from normal (stable) and unstable measurements are shown in Figures S10 and S11.

#### 2.4.4. Results of PPG Data Selection

We evaluated whether the subject’s state changed during the measurement using HRV analysis. As a result, some subjects’ data were considered stable throughout the 10-min experiment, while others exhibited significant changes during recording. For the subsequent analysis, only data where the subject’s state remained unchanged and which were of good quality were included. Therefore, data from 10 out of 18 subjects were used in this study. To improve the reliability of the results with a small sample size, subjects’ age, health status, and measurement stability were standardized.

The experiment was set to last 10 min, assuming subjects could stay in the same resting state at the beginning of the experiment. However, individual differences exist in how long they can remain in a stable, or stationary, resting state. Thus, for some data, such as shown in Figure S11, the resting state changed during the experiment. In these cases, the subject’s state shifted significantly after more than 300 s from the start of the experiment. Therefore, 300 s, which is the standard duration used in this study as mentioned above, may be viewed as the time a person can stay in a resting state in a sitting position, considering individual differences.

Therefore, based on this HRV analysis, 300 s can be considered a good standard value for comparison when adjusting the length of the time series.

## 3. Analysis Methods

### 3.1. Reconstruction into a Delay Coordinate System

In many real-world observations, it is impossible to observe all state variables of a nonlinear dynamical system at the same time; therefore, delay coordinate system reconstruction is used in nonlinear time series analysis to recreate the original system from the acquired time series [29,69]. This reconstruction method is based on Takens’ embedding theorem and aims to reproduce the system’s multidimensional nature and reveal its hidden dynamical structure. Because it is generally believed that all state variables of the pulse wave system cannot be observed, and only one-dimensional data can be obtained from PPG, reconstruction to a delay coordinate system was performed [11,25,26]. The process transforms the observed one-dimensional, *n*-point-long time series data *x*(*t*) into an *m*-dimensional vector *v*(*t*) as described by Equation (3).

(3) $$v\left(t\right)=\left(x\left(t\right),x\left(t+\tau \right),\ldots ,x\left(t+\left(m-1\right)\tau \right)\right),$$

where *τ* is the time delay value, *m* is the reconstruction dimension, and *t* = 1, 2, 3, …, *N* (*N* = *n* − $\left(m-1\right)\tau$).

The time delay value is often determined by autocorrelation [29]. Generally, if the time delay is too small, the correlation will be extremely high; therefore, an appropriate setting is necessary. In this study, the autocorrelation value is calculated, and the time when it first falls below *1 − 1*/*e* is used [41].

Next, the reconstruction dimension is determined by the false neighborhood method [29]. This allows us to find the dimension in which the percentage of points that were neighbors in the (*m* − 1)-dimensional space and are no longer neighbors in the *m*-dimensional space is close to zero.

### 3.2. Recurrence Plot (RP)

Recurrence plot (RP) visualizes the distance relationship between points on the attractor and is used for detecting dynamical behavior of time series [31,32,33]. RP is a two-dimensional binary image with a length of the total number of points on the attractor, *N*, and a matrix is created by Equation (4):

(4) $$R_{i,j}\left(\varepsilon \right)=\left\{\begin{array}{c} 0,\;if\;\left\|v(i)\;-\;v(j)\right\|>\;\varepsilon \\ 1,\;if\;\left\|v(i)\;-\;v(j)\right\|<\;\varepsilon \; \end{array}\right.$$

where $R_{i,j}$ is *i*,*j* th pixel on RP, *ε* is the threshold value, and *i*,*j* are 1, 2, 3, …, *N*. In this study, the threshold value is set at a value where the recurrence rate (*RR*) of the recurrence plot obtained by Equation (5) is close to 10% [31]:

(5) $$RR\left(\varepsilon \right)=\frac{1}{N^{2}}\sum_{i,j=1}^{N}R_{i,j}\left(\varepsilon \right).$$

### 3.3. Recurrence Quantification Analysis (RQA)

RQA can extract quantitative features from the RP, and although there are various methods, in this study, we calculated four values that characterize the diagonal lines [31,33]. These indices are important for quantitatively assessing the system’s regularity and chaos and are appropriate for analyzing the dynamics of time series, which is the objective of this study.

*D*(*l*) is the number of diagonal lines of length *l* defined by Equation (6). The RQA will be performed based on this value.

(6) $$D\left(l\right)=\left(1-R\left(i-1,j-1\right)\right)\left(1-R\left(i+l,j+l\right)\right)\prod_{k=0}^{l-1}R\left(i+k,j+k\right)$$

The first RQA index is determinism (*DET*), which is the ratio of points forming a diagonal line, as defined by Equation (7). When a time series is deterministic, the *DET* value tends to be close to 1 [31]. Determinism means that the system is not created randomly but is driven by some rule.

(7) $$DET=\frac{\sum_{l\geq 2}l\left|D\left(l\right)\right|}{\sum_{l\geq 1}l\left|D\left(l\right)\right|}.$$

The second index is $L_{max}$. The orbit instability, or exponential orbit divergence, of a chaotic time series can be estimated as the inverse of the longest diagonal in the RP, $L_{max}$, defined by Equation (8). Short $L_{max}$ indicates complex dynamics with rapid divergence, while longer values tend to indicate periodic behavior [31]. It is also considered to be inversely proportional to the Lyapunov exponent [31].

(8) $$L_{max}=\max\left\{l|D\left(l\right)\neq \emptyset \right\}.$$

The third index is *L*. The average prediction time of an attractor is estimated by the average length of the diagonal line, *L*, which is calculated using Equation (9) [31]. The average prediction time indicates how long the system is predictable, and a long *L* suggests that the system is more regular.

(9) $$L=\frac{\sum_{l\geq 2}l\left|D\left(l\right)\right|}{\sum_{l\geq 2}\left|D\left(l\right)\right|}$$

The fourth measure is entropy (*ENTR*), calculated using Equation (10). *ENTR* is defined as the probability of finding a diagonal line of length *l* in the RP, as shown in Equation (11) [31]. The higher the *ENTR* value, the more complex the system [31].

(10) $$ENTR=-\sum_{l\geq 2}p\left(l\right)\log p\left(l\right)$$

(11) $$p\left(l\right)=\frac{\left|D\left(l\right)\right|}{\sum_{l\geq 2}\left|D\left(l\right)\right|}$$

### 3.4. Error

The relative error, $E_{l}$, for each of the above four RQA values, *S*, is determined by Equation (12) for a given time series length, *l*, and a reference time series length, *T* = 300 s, which choice is discussed in Section 2.4.4.

(12) $$E_{l}=\frac{\left|S_{l}-S_{T}\right|}{S_{T}}\times 100\%$$

## 4. Results

### 4.1. PPG Time Series Subsets

**Figure 6:**
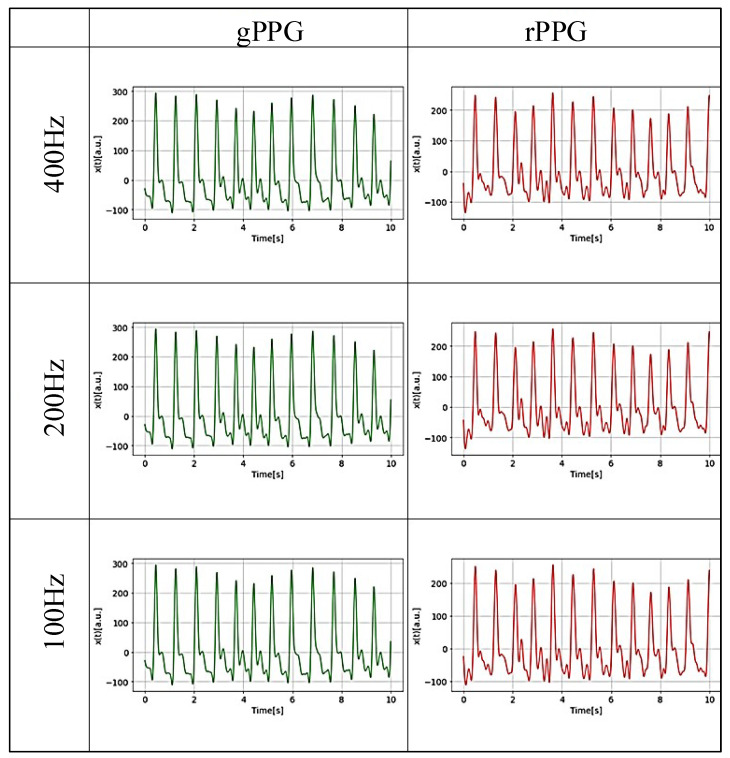
An example of the PPG waveform of the PPG data (10 s) at sampling frequencies of 400 Hz, 200 Hz, and 100 Hz.

The choice of time series starting point may affect the analysis results; therefore, in this study, to proceed with analysis, subsets were created by varying the length of the preprocessed data every 10 s from 10 s (more than 10 heartbeat cycles) to ~200 s, with the initial position of the data shifted every 30 s. A reference data set for 300 s was also created in the same way. These were created for rPPG and gPPG at each frequency. As an example of the PPG data obtained, Figure 6 shows 10 s of rPPG and gPPG at each investigated frequency for the same person after pretreatment. Inspection of the waveforms of the PPG data reveals that no significant information loss occurred as the sampling frequencies varied in this study. However, when comparing gPPG and rPPG, differences can be seen in the waveforms.

### 4.2. Parameter Settings

For each PPG time series, we set parameters, reconstruct the delay coordinate system, create RPs, and perform RQA. First, as parameter settings, we obtain the time delay value *τ* and the reconstruction dimension *m* required for reconstruction to the delay coordinate system. The results of the autocorrelation function calculation and the false neighborhood method are shown in Figures S12–S15. These figures summarize the results for 10, 60, and 120 s at each sampling frequency.

First, in the autocorrelation graph, as the time series length increases, only a slight change can be observed. This applies to all data, each having its own specific value. The variation with sampling frequency indicates that when the frequency is halved or quartered, the autocorrelation decreases correspondingly by the same factors.

Next, when analyzing the results of the false neighborhood method with longer time series, the false neighborhood ratio decreased to zero as the dimension increased from 4 to 5 at 10 s, indicating that four dimensions are sufficient. At 60 and 120 s, increasing the dimensions from 5 to 6 also led to a zero-error neighborhood ratio, suggesting that five dimensions are sufficient. This pattern remained even when the sampling frequency was lowered. The findings were similar for gPPG and rPPG.

### 4.3. Reconstructed Attractor and Recurrence Plot

Using the parameters obtained above, the reconstruction to the delay coordinate system and RP calculation were performed. The RP threshold was determined based on the *RR*, as described above, and was approximately 0.1 times the maximum distance between points of the reconstructed attractor.

**Figure 7:**
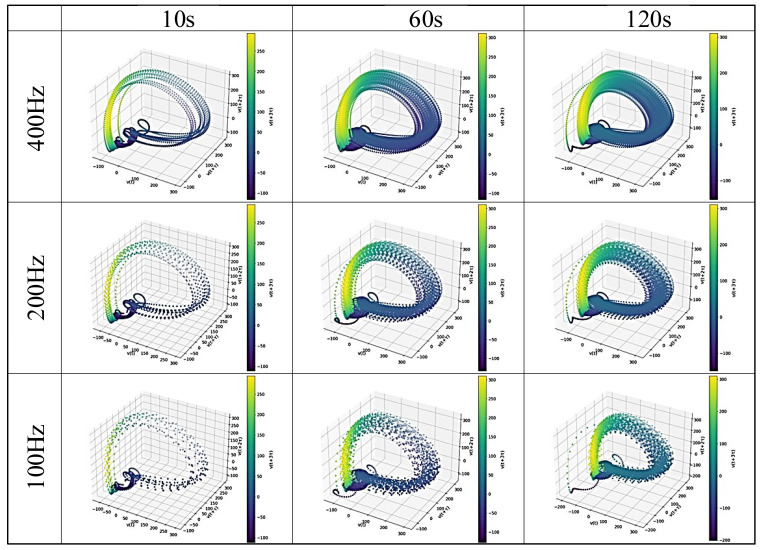
An example of the reconstructed attractor for the gPPG data subsets.

**Figure 8:**
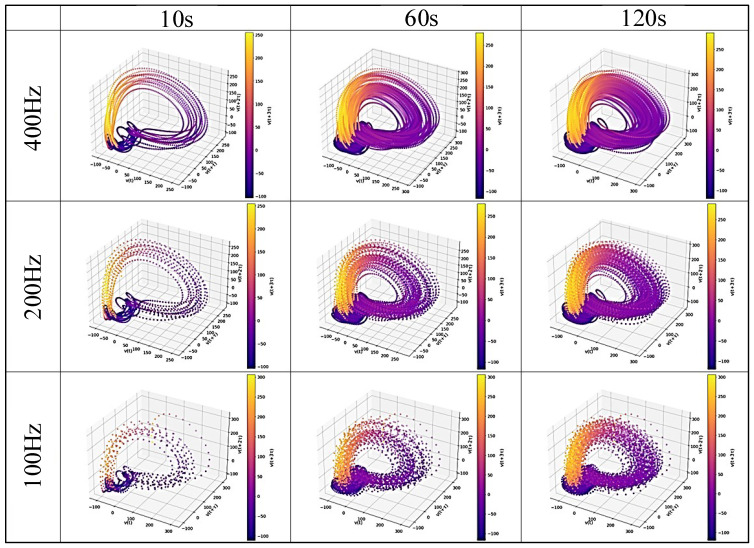
An example of the reconstructed attractor for the rPPG data subsets.

**Figure 9:**
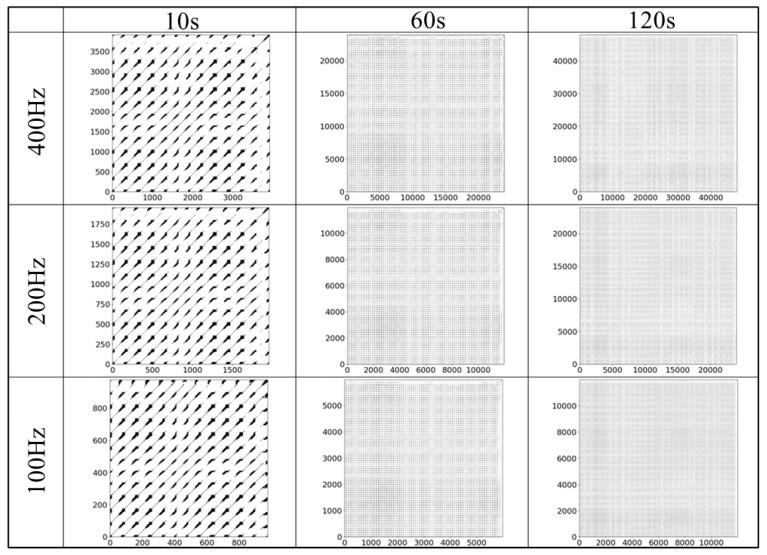
An example of the RP for the gPPG data subsets.

**Figure 10:**
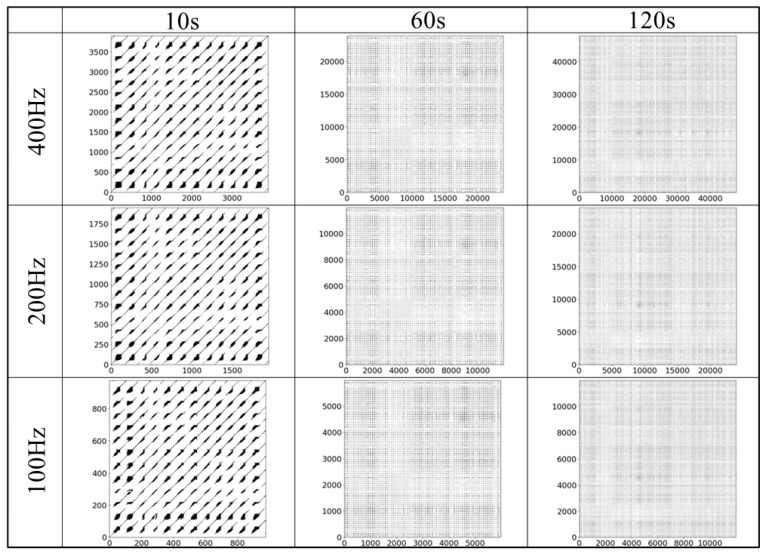
An example of the RP for the rPPG data subsets.

Figure 7, Figure 8, Figure 9 and Figure 10 display examples of the reconstructed attractors and RPs using the same subject’s data. The reconstructed attractor was 5-dimensional as described in Section 4.2, but for visualization purposes, only up to four dimensions are shown here, with colors indicating the values in the fourth dimension.

The reconstructed attractors (Figure 7 for gPPG and Figure 8 for rPPG) display a complex yet recurring behavior. These attractors make it easier to observe the dependence on frequency differences, which cannot be seen in the raw PPG data. Comparing the attractors of gPPG and rPPG, their shapes appear similar, but they do not behave exactly the same, indicating differences caused by the wavelength of light and noise effects from measurement equipment. To analyze these features in detail, RPs (Figure 9 for gPPG and Figure 10 for rPPG) visualizing neighborhood relationships were created, and RQA was conducted.

### 4.4. Recurrence Quantification Analysis

**Figure 11:**
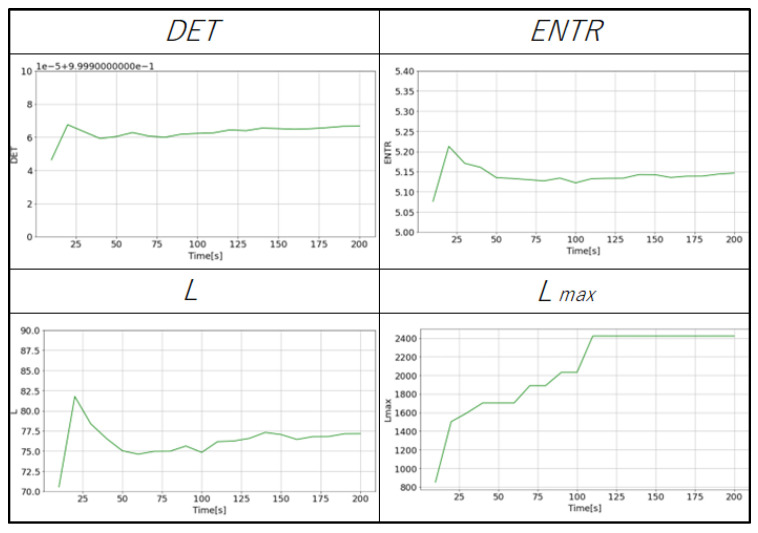
An example of the variation of the RQA indexes of the gPPG (400 Hz with 200 s time series length).

**Figure 12:**
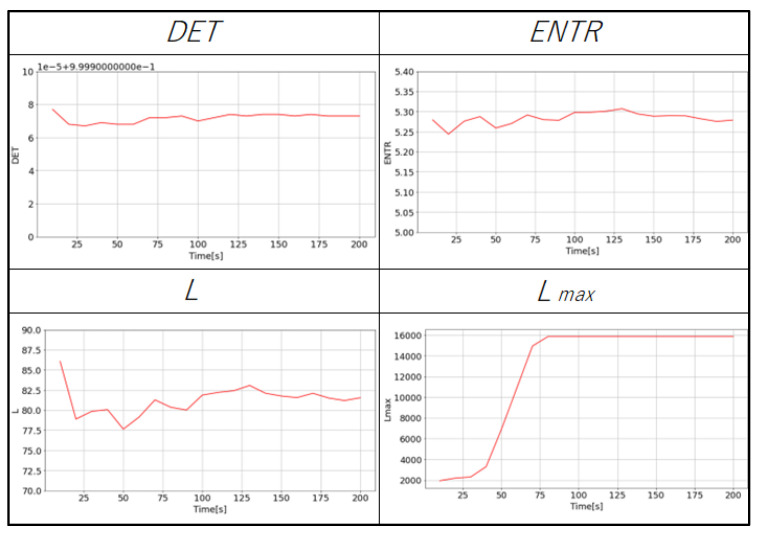
An example of the variation of the RQA indexes of the rPPG (400 Hz with 200 s time series length).

The RQA is used to quantify the dynamical characteristics from the RPs. Figure 11 (gPPG) and Figure 12 (rPPG) show the changes in the values of each RQA result for the same subject. The changes in *DET*, *L*, and *ENTR* become more stable as the length of the time series increases. The value of $L_{max}$ for gPPG and rPPG stopped changing and remained constant when the time series length became longer. The results of gPPG and rPPG are not the same, especially the values of $L_{max}$ saturate faster for the rPPG.

### 4.5. Effects of Down-Sampling

**Figure 13:**
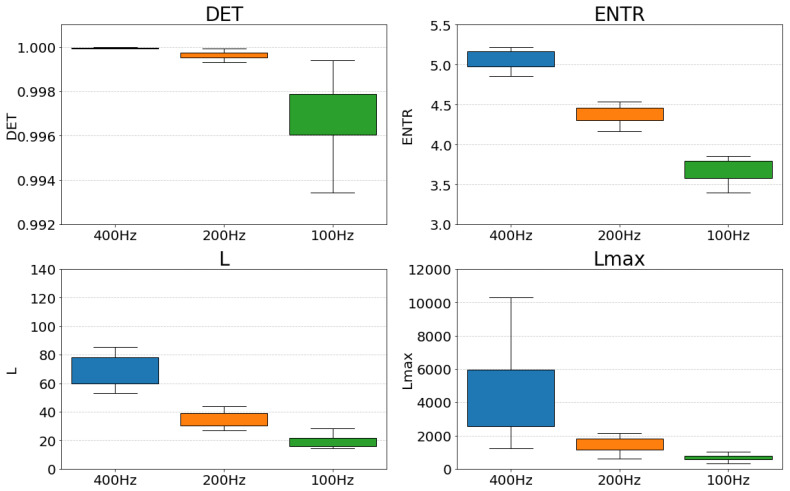
Distribution of RQA indices at each frequency for gPPG.

**Figure 14:**
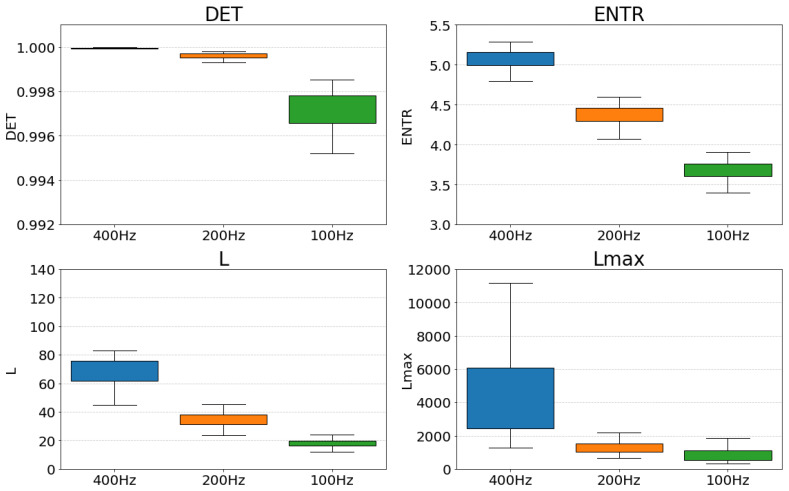
Distribution of RQA indices at each frequency for rPPG.

First, we compare the effect of down-sampling at each frequency. Figure 13 and Figure 14 show a box-and-whisker plot of the RQA index values for each frequency (400 Hz, 200 Hz, and 100 Hz). The results show that all the indices show a decreasing trend when the frequency is lowered. A closer look at the effect of decreasing frequency shows that *DET* shows an increase in the variability of values, *ENTR* shows an overall decrease while the variability of values remains the same, and *L* and $L_{max}$ show a decrease in the variability of values and a more coherent distribution. Comparing the results for gPPG and rPPG, we can see that they show similar distributions.

In this study, we ranked the indicators at each frequency and calculated correlation coefficients between the down-sampled results and the rankings. Spearman’s rank correlation test was used with a 95% confidence level. The results showed that statistically significant rank correlations existed for all data. The correlation coefficients are shown in Table 2.

The correlation coefficients indicate a strong overall correlation and show that down-sampling did not significantly alter the relative relationships. Specifically, for L, ENTR, and DET, there was a consistently strong positive correlation across all frequency combinations. However, for both gPPG and rPPG, the correlation at $L_{max}$ compared to 400 Hz suggests it is weaker than for the other indices. Nonetheless, 200 Hz and 100 Hz still exhibit strong correlations, similar to the other indices. This suggests that down-sampling must be approached cautiously if higher frequencies are accurate, but it is also possible that the 400 Hz setting introduces excess information when generating the RPs, leading to values that differ from the original. Examining the distribution of $L_{max}$ in Figure 13 and Figure 14, the data are too dispersed to reflect the same resting state. Therefore, the overall conclusion is that the relative position remains largely unchanged after down-sampling, and 200 Hz may be suitable considering the change in the distribution of the data.

### 4.6. Difference Between gPPG and rPPG by Recurrence Quantification Analysis

In the previous section, it was suggested that gPPG and rPPG may have similar distributions based on the box-and-whisker diagram. In this section, a Wilcoxon signed-rank test was additionally performed on the dataset with paired data for each subject. This is because the Shapiro–Wilk test did not confirm the normal distribution of each RQA index. The *p*-value results are summarized in Table 3, with significant differences highlighted.

The results show that there are no significant differences except for *L* and $L_{max}$ at 100 Hz, indicating that there are basically no significant differences between the values obtained by RQA for gPPG and rPPG at 400 Hz and 200 Hz. However, since the data at 100 Hz showed significant differences in the two indices, down-sampling to 100 Hz should be avoided because some information would be lost and a difference would be generated in the data where originally was no difference between gPPG and rPPG. However, the results in 4.5 show that there is a strong correlation with the 200 Hz data, and therefore, if one does not pay attention to the difference between gPPG and rPPG, there should be no particular problem in handling the data.

Combined with the results of Section 4.5, it can be concluded that a sampling frequency of 200 Hz is suitable for handling different types of PPG because it reduces the variability of all RQA indices and does not create significant differences between rPPG and gPPG.

### 4.7. Effects of Time Series Length

**Figure 15:**
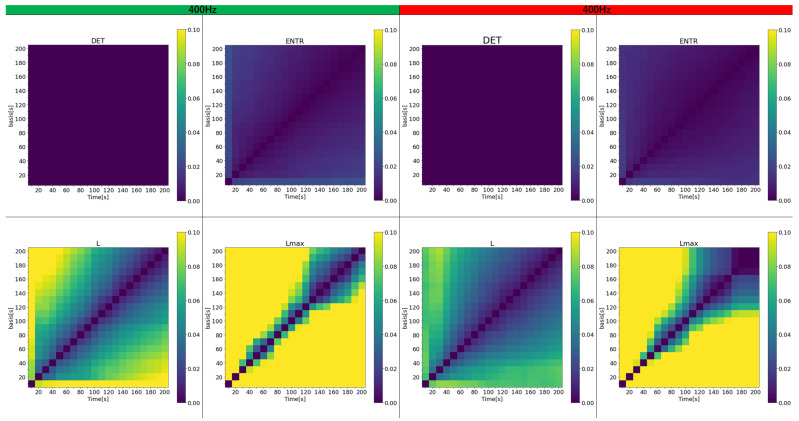
Color map of the average error for variable time series length for PPG (400 Hz): (**left half**) gPPG; (**right half**) rPPG.

**Figure 16:**
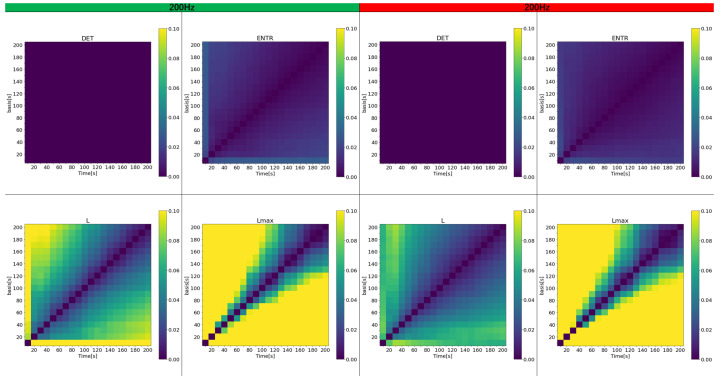
Color maps of the average error for variable time series length for PPG (200 Hz): (**left half**) gPPG; (**right half**) rPPG.

**Figure 17:**
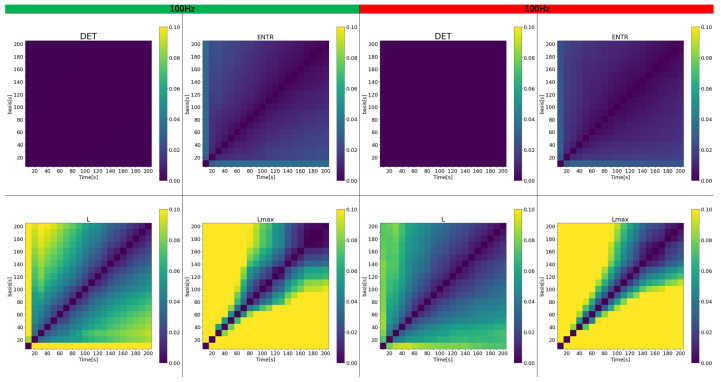
Color maps of the average error for variable time series length for PPG (100 Hz): (**left half**) gPPG; (**right half**) rPPG.

Next, the effect of time series length is examined. To represent a more realistic and general situation where the reference value is unknown or difficult to estimate, first, all data were analyzed with time series lengths ranging from 10 to 200 s. The error was then calculated between the results for various time series lengths and the reference value obtained within the same 10 to 200-s range. Figure 15, Figure 16 and Figure 17 display a color map of the errors at 400 Hz, 200 Hz, and 100 Hz, respectively. The horizontal axis represents the varied time series lengths, while the vertical axis compares different lengths. For clarity, only error values from 0 to 0.1 (0% to 10%) are color-coded.

The results show that the error for *DET* is nearly zero in all cases and remains largely unaffected by the length of the time series. In contrast, the error for *ENTR* is generally low but increases slightly as the length of the compared time series grows. *L* exhibits a higher error than the previous two, and this error escalates as either the length of the time series or the reference time series increases, shown by the green and yellow areas. The rPPG results feature fewer yellow areas, although the impact of frequency is less noticeable. For $L_{max}$ both gPPG and rPPG, the error is very high and increases even when the time series length differs by 50 s from the length being compared. Across all results, the region of lower error, marked by the purple area, expands as the length of the investigated and reference time series increases. As the frequency decreases, the high-error area (represented by yellow) shrinks, and the error drops. While gPPG and rPPG results are quite similar, the 400 Hz data show that rPPG has slightly smaller yellow areas.

### 4.8. Error for gPPG with the Standard Reference Value (300 s)

**Figure 18:**
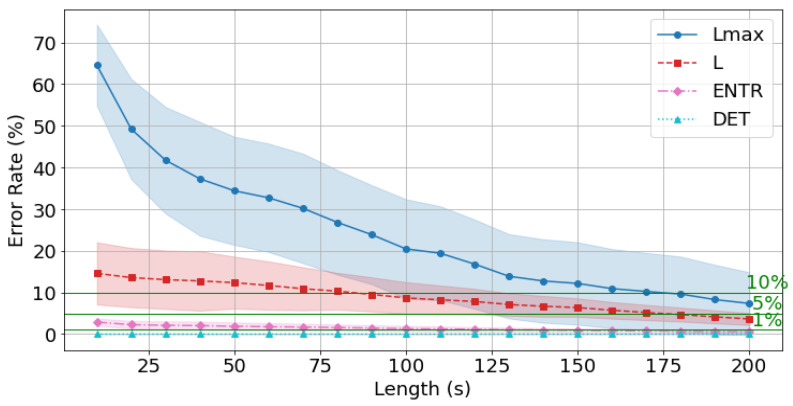
Change in the average error rate (±0.5σ) of gPPG (400 Hz) from the standard reference value.

**Figure 19:**
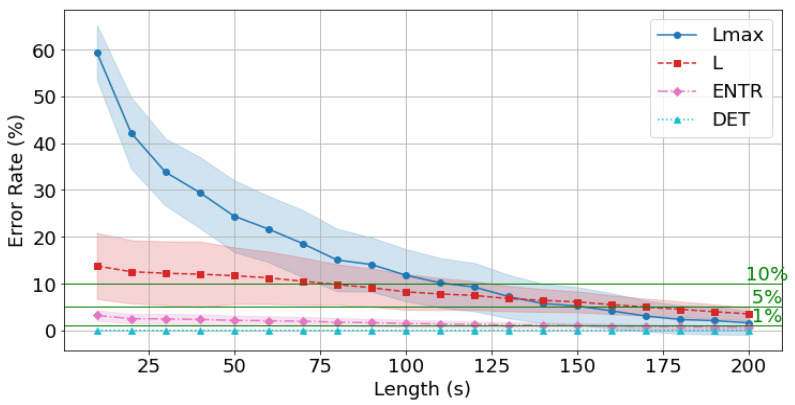
Change in the average error rate (±0.5σ) of gPPG (200 Hz) from the standard reference value.

**Figure 20:**
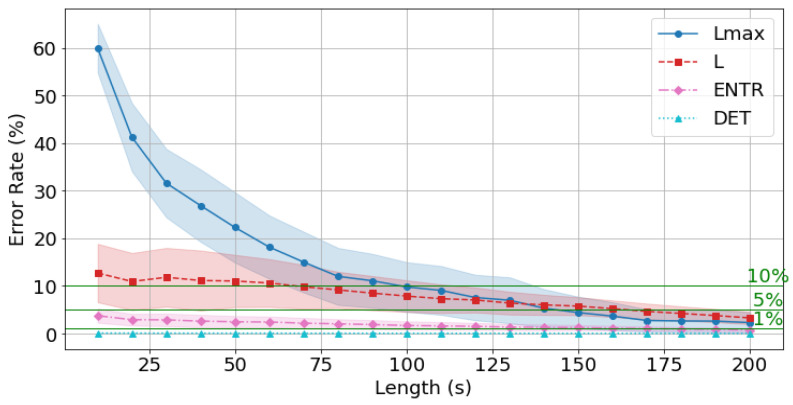
Change in the average error rate (±0.5σ) of gPPG (100 Hz) from the standard reference value.

From this point onward, the error is calculated using the reference value (300 s) for all data and is compared with the time series length. First, the gPPG results are shown in Figure 18, Figure 19 and Figure 20 and Table 4. The results show that as the length of the time series increases, the error rate tends to decrease for all of the RQA indices. In terms of the degree of decrease, the error rates for *DET* and *ENTR* are quite low even at short time series lengths, and then they gradually decrease. *L* drops from approximately 15% to 3%. $L_{max}$ has a high error rate at short time series lengths, but this rate decreases significantly as the time series length increases. This trend was also observed across different frequencies.

The average error rate for *DET* + 0.5σ (where σ is the standard deviation) was less than 1% at 10 s, and the higher the frequency, the lower the error rate. The average *ENTR* + 0.5σ 1% cutoff was 180 s at 400 Hz (Figure 18), 170 s at 200 Hz (Figure 19), and 170 s at 100 Hz (Figure 20), and the lower the frequency, the lower the cutoff. The $L_{max}$ 5% cutoff was not reached at 400 Hz, but achieved at 200 Hz for 160 s and at 100 Hz for 150 s.

The standard deviations of the error rates for *all* RQA indices were smallest at 200 s. For the *DET*, *ENTR*, and *L* indices, standard deviations were large at short time series lengths and then decreased, while for the $L_{max}$, it was small at short time series lengths, then increased, and finally significantly decreased reaching a minimal value. This trend was also observed for different frequencies. A closer look at the effect of frequency shows that *DET* and *ENTR* become smaller as the frequency increases, while *L* and $L_{max}$ become smaller as the frequency decreases.

The errors from the reference value (300 s) for *all* RQA indices were less than 10% for 180 s at 400 Hz, 120 s at 200 Hz, and 100 s at 100 Hz, and less than 5% for 170 s at 200 Hz, and 170 s at 100 Hz, respectively.

### 4.9. Error for rPPG with the Standard Reference Value (300 s)

**Figure 21:**
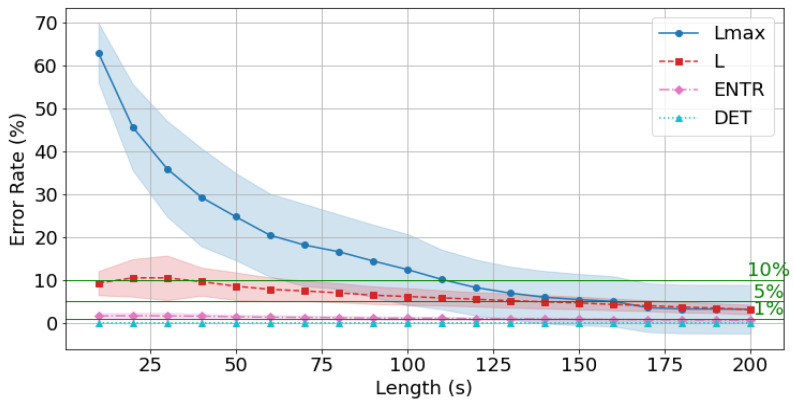
Change in the average error rate (±0.5σ) of rPPG (400 Hz) from the standard reference value.

**Figure 22:**
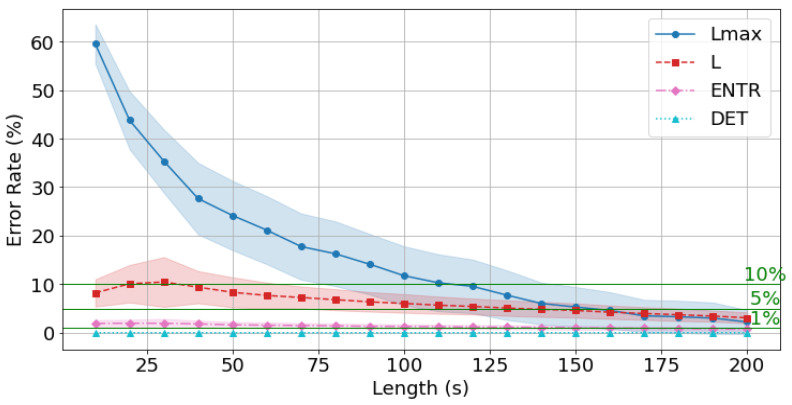
Change in the average error rate (±0.5σ) of rPPG (200 Hz) from the standard reference value.

**Figure 23:**
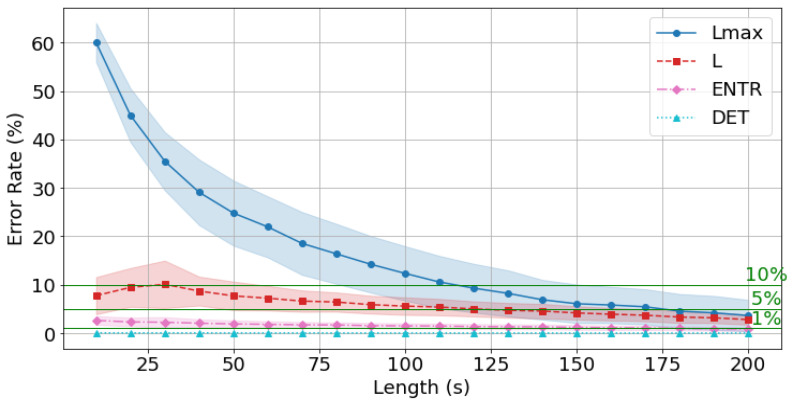
Change in the average error rate (± 0.5σ) of rPPG (100 Hz) from the standard reference value.

The results of rPPG are shown in Figure 21, Figure 22 and Figure 23 and Table 5. The results show a similar trend to that of gPPG, with the difference being that the mean error rate and standard deviation of *L* were higher at shorter times and then decreased, while the mean error rate and standard deviation of the other RQA indices were similar. However, the mean error rate at 200 s was lower for rPPG for *DET* and $L_{max}$, and lower for gPPG for *ENTR* and *L.* The standard deviation trends were similar.

As seen in Figure 21, Figure 22 and Figure 23, the average error rate for *DET* + 0.5σ was below 1% at 10 s, and the higher the frequency, the lower the error rate. The *ENTR* was under 5% starting from 10 s for all frequencies. The *L* below 10% was achieved at 40 s for 400 Hz, at 40 s for 200 Hz, and at 40 s for 100 Hz. It dropped below 5% at 140 s for 400 Hz, 150 s for 200 Hz, and 130 s for 100 Hz. The $L_{max}$ results show that the 10% cutoff at 400 Hz, 200 Hz, and 100 Hz was 120 s, and the 5% cutoff was 170 s at 400 Hz and 200 Hz, and 180 s at 100 Hz, with lower frequencies having lower cutoffs.

The rPPG’s error rate from the standard value (300 s) is less than 10% for *all* the RQA indices: 120 s for 400 Hz, 200 Hz, and 100 Hz, and less than 5% for 170 s for 400 Hz and 200 Hz, and 180 s for 100 Hz.

## 5. Discussion

The purpose of this study was to explore data reduction methods for PPG data that would minimize the data used, yet be sufficient for robustly extracting its complex dynamical features through nonlinear time series analysis, represented here by RQA. Although the impact of down-sampling could not be directly assessed from the PPG waveforms, it was possible to observe the effects of down-sampling and changes in the length of the time series by reconstructing the data into a delay coordinate system (Figure 7 and Figure 8) and generating RPs (Figure 9 and Figure 10). Subsequently, RQA was performed to analyze RPs and compare the data characteristics.

First, the results of the down-sampling effect demonstrated in Figure 13 for gPPG and Figure 14 for rPPG showed a decrease in the overall distribution of values for all RQA indices as the frequency decreased. However, for *DET*, the range of the distribution widened, and for *L* and $L_{max}$, the distribution clumped together. Furthermore, as seen in Table 2, a rank correlation check on whether the relative position of each value had changed revealed that correlations existed for all indicators. For the $L_{max}$ correlation was weaker compared with the *L*. It might be related to the fact that the distribution of the data at 400 Hz was considerably wider than that of the others (Figure 13 and Figure 14), suggesting that the data were too scattered, even though they were from the same resting state, and thus may have contained excessive information in creating the RPs. From the changes in the overall distribution of the indices and the results of the rank correlation, it can be inferred that 200 Hz is an optimal and sufficient sampling frequency for PPGs.

Next, the effect of time series length was verified by comparing the results of 10 to 200 s with each other and with the standard value of 300 s. As seen in Figure 18, Figure 19 and Figure 20 and Table 4 for gPPG and Figure 21, Figure 22 and Figure 23 and Table 5 for rPPG, the results for the 10 to 200 s time series showed that *DET* had low errors for all time series lengths and was not affected by it. At the same time, *ENTR* had low errors as well and was only minimally affected by the time series length. *L*, and $L_{max}$ tended to increase with distance from the compared time series length and to decrease with longer time series length, showing that the error was considerably affected by time series length. The results also showed that the error became lower as the frequency was lowered. For *DET* and *ENTR,* the errors were low starting at 10 s, and for *L* and $L_{max}$ the errors were high at shorter time-series lengths, and the errors decreased as the time-series length increased. When the frequency was lowered, *DET* and *ENTR* showed an increase in error, while *L* and $L_{max}$ showed a decrease in error. In the 200 Hz results (Figure 19 for gPPG and Figure 22 for rPPG), the error rate for all values is less than 5% at 170 s, and the standard deviation is not high, so we suggest using the 170-s data as a sufficient time series length rather than the 300-s data. Although we have proposed an error rate of 5% here, the acceptable error rate can be more clearly suggested by checking the difference in each index using the RQA, for example, by comparing the results of a subject at rest and under stress, or by comparing the results of a patient with a certain disease. Additionally, when insufficiently long reference time series (300 s) are available, such as in cases of certain conditions causing significant data instability due to hand shaking, etc., it is possible to estimate possible errors relatively to the shorter time series length, as shown in Figure 15, Figure 16 and Figure 17. This may provide a tool for the robustness estimation of dynamical features evaluation in various applied cases.

Additionally, the comparison of these effects between gPPG and rPPG shows that there is no statistical difference in all the RQA indices, and the errors are not much different. It can be seen that there is no significant difference between the two measurement PPG setups.

## 6. Conclusions

Based on the above results, we propose using 200 Hz down-sampling and a 170 s time series length to extract dynamical information from PPGs via RQA, assuming the state remains constant. These results contribute to the optimization of PPG data processing and analysis, on the one hand, and may contribute to improving the energy consumption efficiency of wearable sensors on the other. Therefore, it establishes a basis for data reduction, which is highly beneficial for various applications, such as health monitoring. At the same time, these results suggest the criteria for unifying the PPG data measurement requirements across different PPG sensors. Moreover, the demonstrated data reduction approach may be applicable and beneficial for a wide range of biomedical signals beyond PPGs.

However, as these results were obtained for a highly unified group of subjects it is necessary to take into account that these results may change with a change in the subject’s physical or mental state, measurement site, and age. Therefore, future work should include further validation by comparing results at higher frequencies and assessing the extent to which the RQA index in PPG data changes with a larger number of subjects, under stress loads, and with other variable conditions, including wider age range, measurement site, physical activity and others.

## Figures and Tables

**Table 2:** Spearman’s rank correlation coefficient for RQA indices corresponding to 400 Hz, 200 Hz, and 100 Hz frequency of gPPG and rPPG.

	$L_{max}$	*L*	*ENTR*	*DET*
**gPPG**				
400 Hz vs. 200 Hz	0.78	0.98	0.98	0.98
400 Hz vs. 100 Hz	0.78	0.95	0.93	0.88
200 Hz vs. 100 Hz	0.94	0.95	0.91	0.89
**rPPG**				
400 Hz vs. 200 Hz	0.60	0.98	0.96	0.98
400 Hz vs. 100 Hz	0.50	0.96	0.92	0.96
200 Hz vs. 100 Hz	0.94	0.97	0.94	0.96

**Table 3:** Wilcoxon signed rank test results of RQA indices between gPPG and rPPG.

	$L_{max}$	*L*	*ENTR*	*DET*
400 Hz	p>0.05	p>0.05	p>0.05	p>0.05
200 Hz	p>0.05	p>0.05	p>0.05	p>0.05
100 Hz	p<0.05	p<0.05	p>0.05	p>0.05

**Table 4:** Summary of the average error rate (%) of gPPG RQA results compared with the reference value.

Time	400 Hz				200 Hz				100 Hz			
	*L_max_*	*L*	*ENTR*	*DET*	*L_max_*	*L*	*ENTR*	*DET*	*L_max_*	*L*	*ENTR*	*DET*
10 s	64.557	14.577	2.881	0.002	59.423	13.777	3.202	0.015	59.970	12.723	3.773	0.108
20 s	49.232	13.567	2.256	0.002	42.189	12.545	2.508	0.014	41.224	10.939	2.911	0.101
30 s	41.739	13.067	2.109	0.001	33.824	12.212	2.460	0.013	31.595	11.823	2.884	0.096
40 s	37.282	12.756	2.039	0.001	29.463	12.012	2.347	0.012	26.874	11.144	2.618	0.089
50 s	34.412	12.358	1.878	0.001	24.410	11.690	2.142	0.011	22.293	11.040	2.478	0.086
60 s	32.731	11.671	1.769	0.001	21.657	11.238	2.065	0.011	18.165	10.616	2.426	0.084
70 s	30.191	10.897	1.680	0.001	18.543	10.497	1.950	0.010	15.014	9.827	2.188	0.080
80 s	26.836	10.309	1.572	0.001	15.044	9.795	1.787	0.010	12.017	9.180	2.047	0.076
90 s	23.885	9.525	1.445	0.001	14.085	9.130	1.645	0.009	11.092	8.500	1.890	0.073
100 s	20.458	8.685	1.300	0.001	11.797	8.247	1.476	0.009	9.790	7.878	1.698	0.069
110 s	19.406	8.226	1.230	0.001	10.167	7.792	1.381	0.008	9.072	7.330	1.614	0.066
120 s	16.807	7.829	1.144	0.001	9.258	7.466	1.278	0.008	7.574	7.059	1.514	0.062
130 s	13.878	7.099	1.032	0.001	7.220	6.801	1.174	0.007	7.002	6.398	1.362	0.058
140 s	12.753	6.673	0.976	0.001	5.735	6.415	1.111	0.007	5.322	6.033	1.308	0.054
150 s	12.162	6.375	0.923	0.001	5.221	6.098	1.039	0.006	4.410	5.763	1.250	0.051
160 s	10.919	5.710	0.835	0.001	4.185	5.497	0.953	0.006	3.664	5.233	1.144	0.048
170 s	10.231	5.181	0.767	0.001	3.055	4.989	0.859	0.005	2.747	4.658	1.044	0.044
180 s	9.616	4.690	0.705	0.001	2.322	4.512	0.790	0.005	2.649	4.233	0.959	0.041
190 s	8.279	4.132	0.634	0.001	2.085	3.996	0.712	0.004	2.616	3.781	0.868	0.037
200 s	7.350	3.629	0.541	0.001	1.613	3.529	0.615	0.004	2.304	3.293	0.743	0.033

**Table 5:** Summary of the average error rate (%) of rPPG RQA results compared with the reference value.

Time	400 Hz				200 Hz				100 Hz			
	*L_max_*	*L*	*ENTR*	*DET*	*L_max_*	*L*	*ENTR*	*DET*	*L_max_*	*L*	*ENTR*	*DET*
10 s	62.898	9.272	1.638	0.002	59.245	8.432	1.950	0.009	59.447	7.916	2.651	0.062
20 s	45.576	10.500	1.698	0.001	44.217	10.231	2.009	0.007	44.910	9.541	2.380	0.049
30 s	35.891	10.498	1.640	0.001	35.843	10.497	2.001	0.007	35.216	10.084	2.319	0.053
40 s	29.250	9.613	1.571	0.001	27.330	9.433	1.860	0.007	28.858	8.717	2.115	0.052
50 s	24.730	8.569	1.438	0.001	23.973	8.475	1.688	0.006	24.623	7.822	1.984	0.047
60 s	20.420	7.817	1.329	0.001	21.131	7.783	1.607	0.006	22.021	7.318	1.890	0.044
70 s	18.147	7.454	1.297	0.001	17.512	7.355	1.538	0.006	18.259	6.709	1.775	0.043
80 s	16.584	7.015	1.225	0.001	15.899	6.958	1.467	0.005	15.986	6.567	1.761	0.041
90 s	14.427	6.470	1.137	0.001	13.932	6.526	1.382	0.005	14.020	6.053	1.626	0.039
100 s	12.437	6.158	1.125	0.001	11.744	6.258	1.368	0.005	11.855	5.791	1.584	0.038
110 s	10.135	5.842	1.079	0.001	10.421	5.926	1.309	0.005	10.236	5.624	1.555	0.035
120 s	8.228	5.511	1.009	0.000	9.748	5.662	1.242	0.004	9.083	5.265	1.460	0.033
130 s	6.952	5.186	0.975	0.000	8.094	5.323	1.205	0.004	8.046	4.943	1.399	0.032
140 s	5.982	4.914	0.942	0.000	6.549	5.003	1.157	0.004	6.850	4.740	1.367	0.030
150 s	5.434	4.658	0.873	0.000	5.888	4.765	1.074	0.003	6.099	4.361	1.252	0.027
160 s	5.024	4.308	0.817	0.000	5.238	4.399	1.012	0.003	5.803	4.082	1.178	0.026
170 s	3.562	4.033	0.772	0.000	4.138	4.129	0.963	0.003	5.468	3.858	1.137	0.024
180 s	3.257	3.707	0.714	0.000	3.931	3.862	0.900	0.003	4.644	3.494	1.035	0.022
190 s	3.233	3.455	0.668	0.000	3.544	3.554	0.825	0.002	3.975	3.290	0.950	0.020
200 s	3.203	3.111	0.609	0.000	2.454	3.170	0.754	0.002	3.433	2.912	0.858	0.019

## Data Availability

No data are available.

## Supplementary Materials

The following supporting information can be downloaded at: https://www.mdpi.com/article/10.3390/s25196232/s1, Figure S1: An example of the amplitude spectrum (gPPG). Figure S2: Frequency response of the *n* th order Butterworth filter (highcut = 0.04 Hz, lowcut = 6 Hz, *n* = 1, 2, …,5). The fourth-order filter (red line) was used for PPG signal preprocessing. Figure S3: Example of noise processing for the gPPG signal: (top) before processing; (bottom) after filter processing. Figure S4: Example of noise processing for the rPPG signal: (top) unprocessed; (bottom) filtered. Figure S5: Typical PPG waveform. Figure S6: Example of detecting the maximum peaks. Figure S7: An example of LF/HF of a stable measurement. Figure S8: An example of LF/HF of an unstable measurement. Figure S9: An example of Poincaré plot: (a) 60~180 points; (b) 180~300 points; (c) 300~420 points; (d) 420~540 points. Figure S10: An example of sd2/sd1 obtained from a stable measurement. Figure S11: An example of sd2/sd1 obtained from an unstable measurement. Figure S12: An example of the autocorrelation graph of the gPPG. Figure S13: An example of the autocorrelation graph of the rPPG. Figure S14: An example of dimension estimation by the false neighborhood method for gPPG. Figure S15: An example of dimension estimation by the false neighborhood method for rPPG.

## Abbreviations

The following abbreviations are used in this manuscript:

PPG	Photoplethysmogram
rPPG	Photoplethysmogram recorded with red light
gPPG	Photoplethysmogram recorded with green light
HRV	Heart Rate Variability
RP	Recurrence Plot
RQA	Recurrence Quantification Analysis
