# Supplementary material for: Data Reduction Methodology for Dynamic Characteristic Extraction in Photoplethysmogram
Source: Sensors (Basel). 2025 Oct 8;25(19):6232. doi: 10.3390/s25196232 (PMC12526808; doi:10.3390/s25196232)
Supplement: Supplementary file 1 [file sensors-25-06232-s001.zip › sensors-3826611-supplementary.pdf]

*Supplementary Materials for Article*

# Data Reduction Methodology for Dynamic Characteristic Extraction in Photoplethysmogram

**Nina Sviridova [1,2*] and Sora Okazaki [1]**

[1] Department of Intelligent Systems, Tokyo City University, 1-28-1 Tamazutsumi, Setagaya-ku, Tokyo, 158-8557 Japan

[2] International Research Center for Neurointelligence, The University of Tokyo, 7–3–1 Hongo Bunkyo-ku, Tokyo, 113–0033 Japan

* Correspondence: nina@tcu.ac.jp

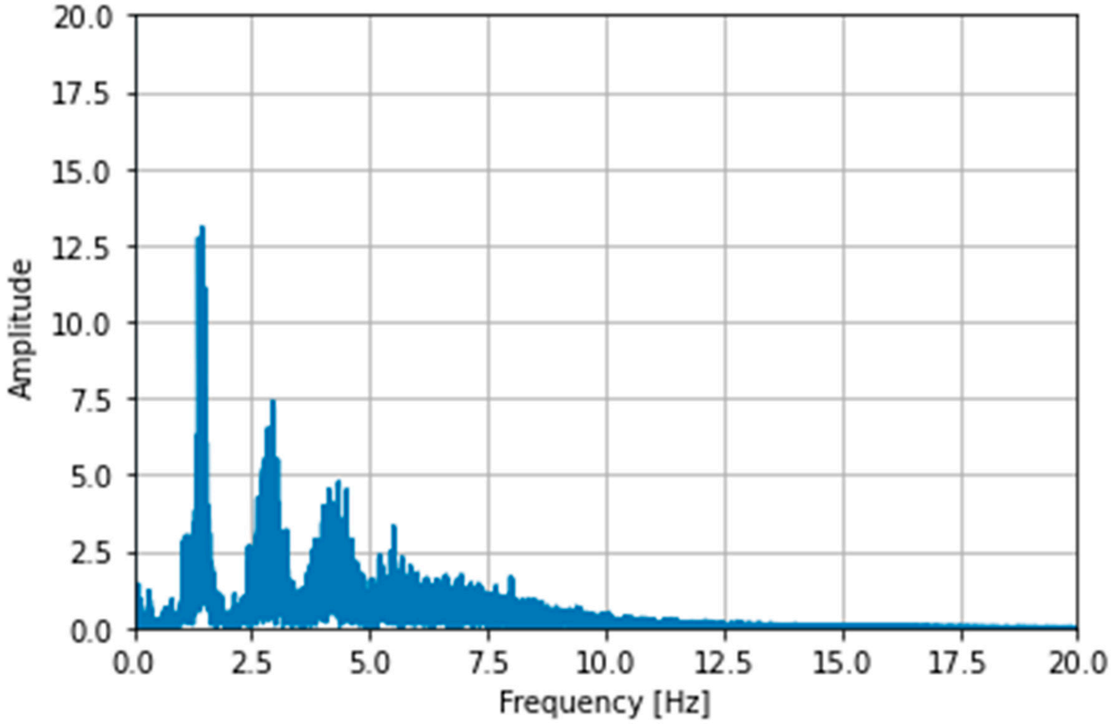


**Figure S1.** An example of the amplitude spectrum (gPPG).

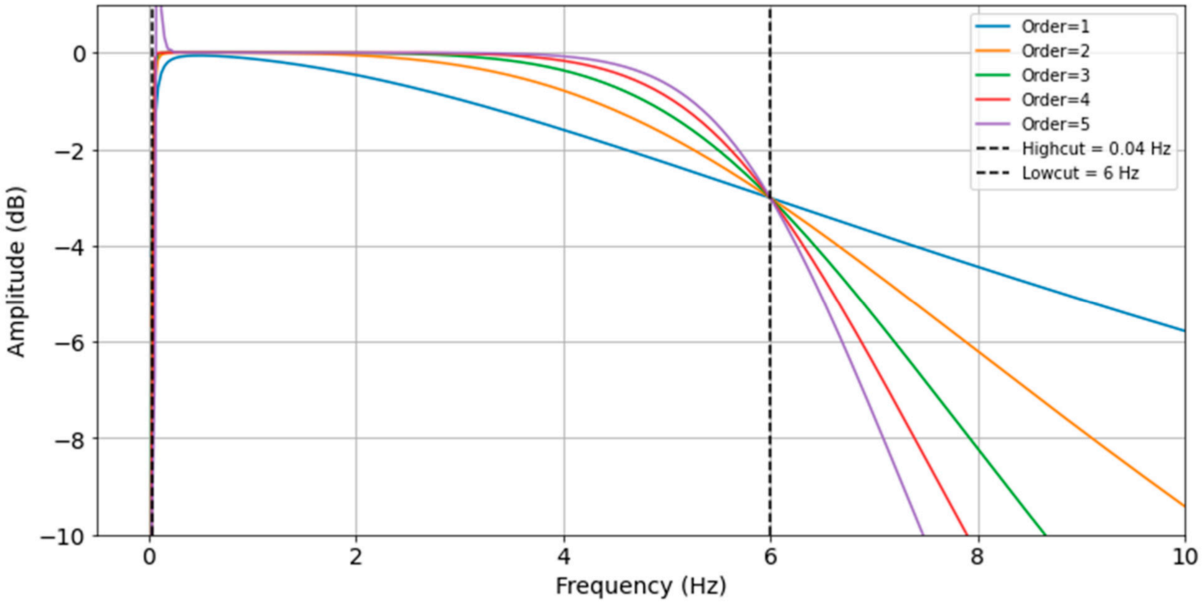


**Figure S2.** Frequency response of the $n^{th}$ order Butterworth filter (highcut = 0.04Hz, lowcut = 6Hz, $n$=1, 2, …,5). The fourth-order filter (red line) was used for PPG signal preprocessing.

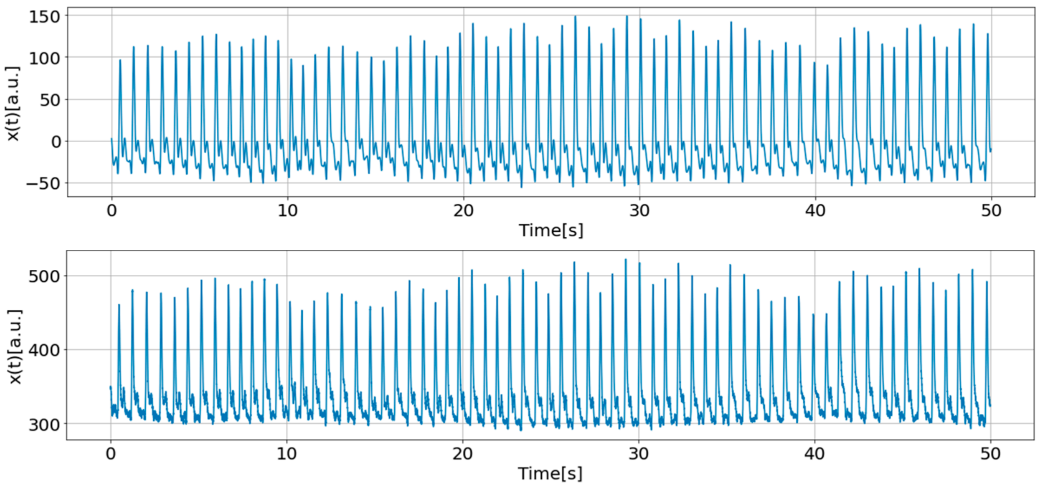

**Figure S3.** Example of noise processing for the gPPG signal: (**top**) before processing; (**bottom**) after filter processing.

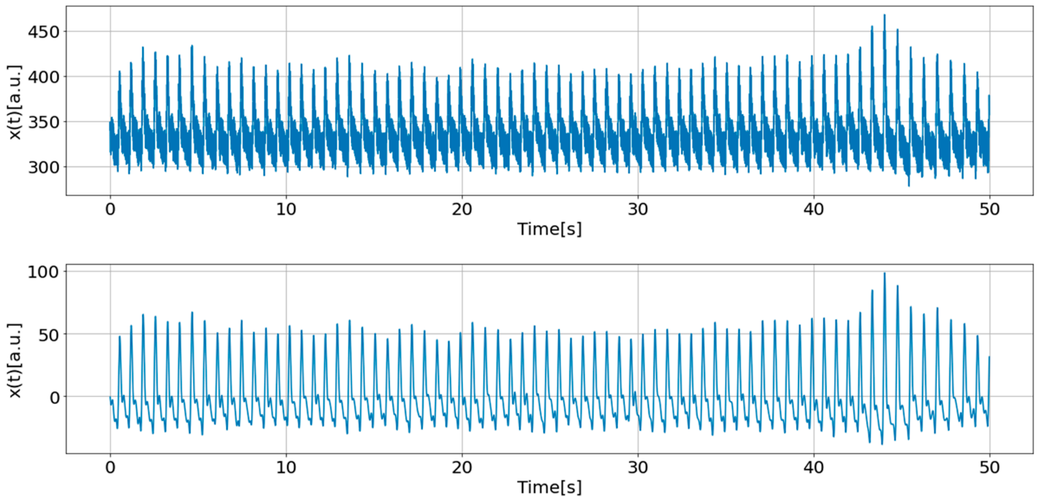

**Figure S4.** Example of noise processing for the rPPG signal: (**top**) unprocessed; (**bottom**) filtered.

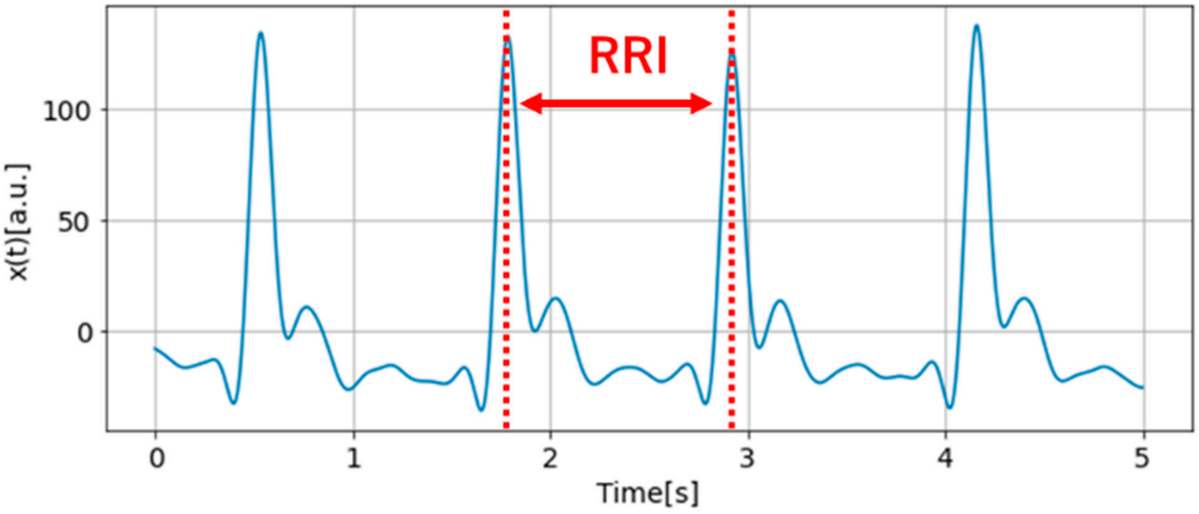


**Figure S5.** Typical PPG waveform.

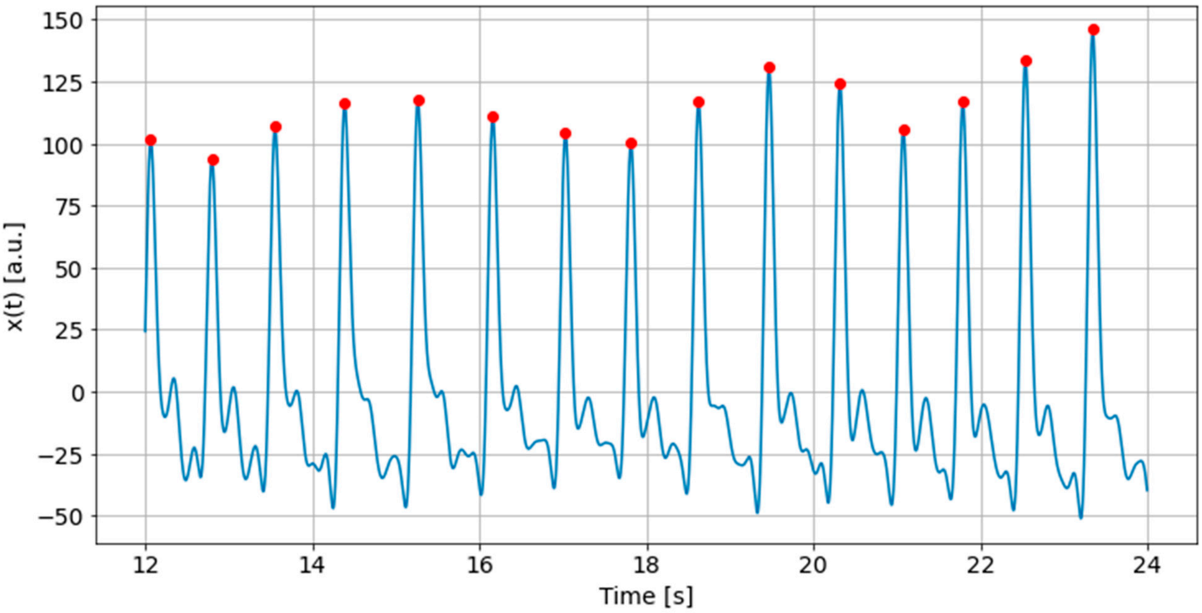


**Figure S6.** Example of detecting the maximum peaks.

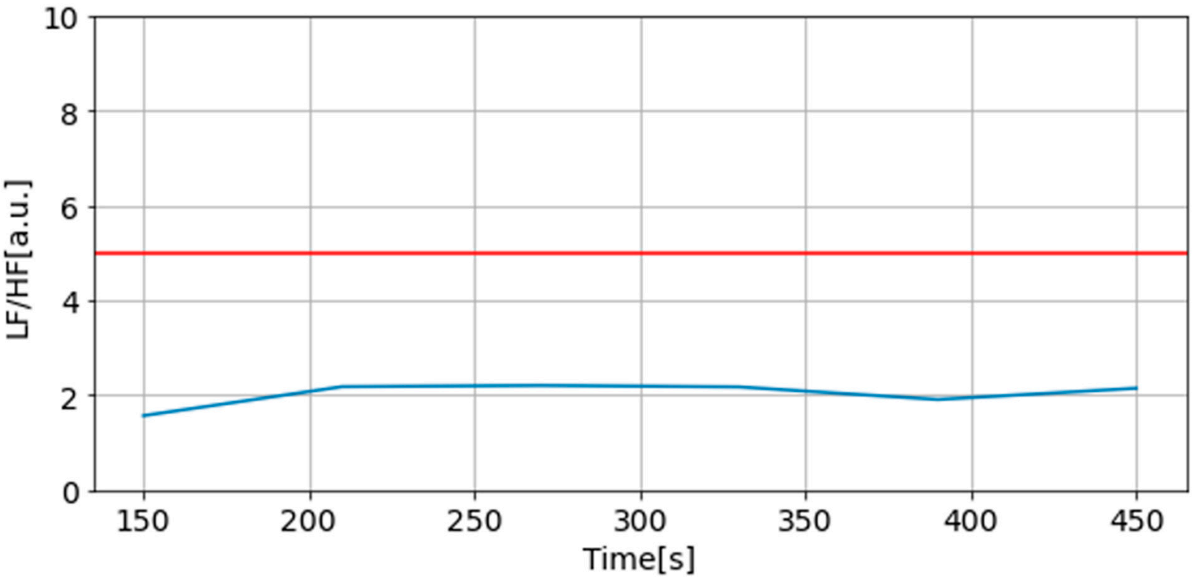


**Figure S7.** An example of the LF/HF of a stable measurement.

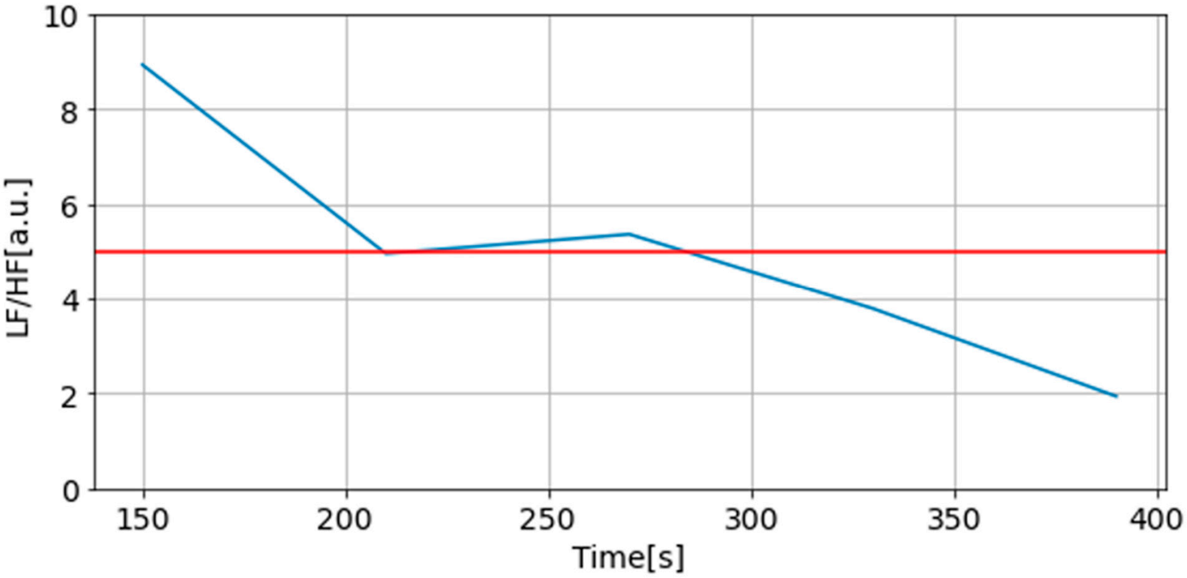


**Figure S8.** An example of the LF/HF of an unstable measurement.

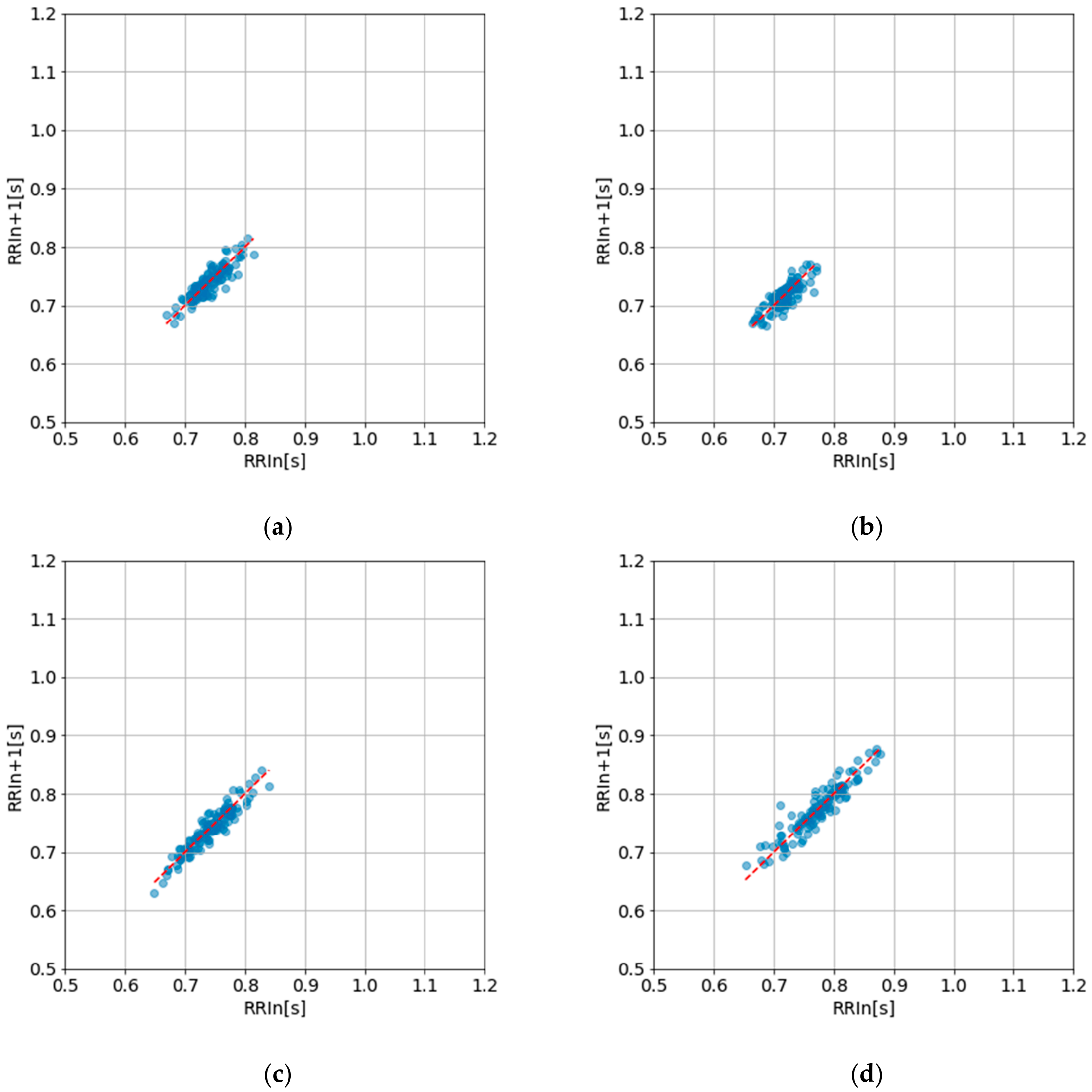


**Figure S9.** An example of a Poincaré plot: (**a**) 60~180 points; (**b**) 180~300 points; (**c**) 300~420 points; (**d**) 420~540 points.

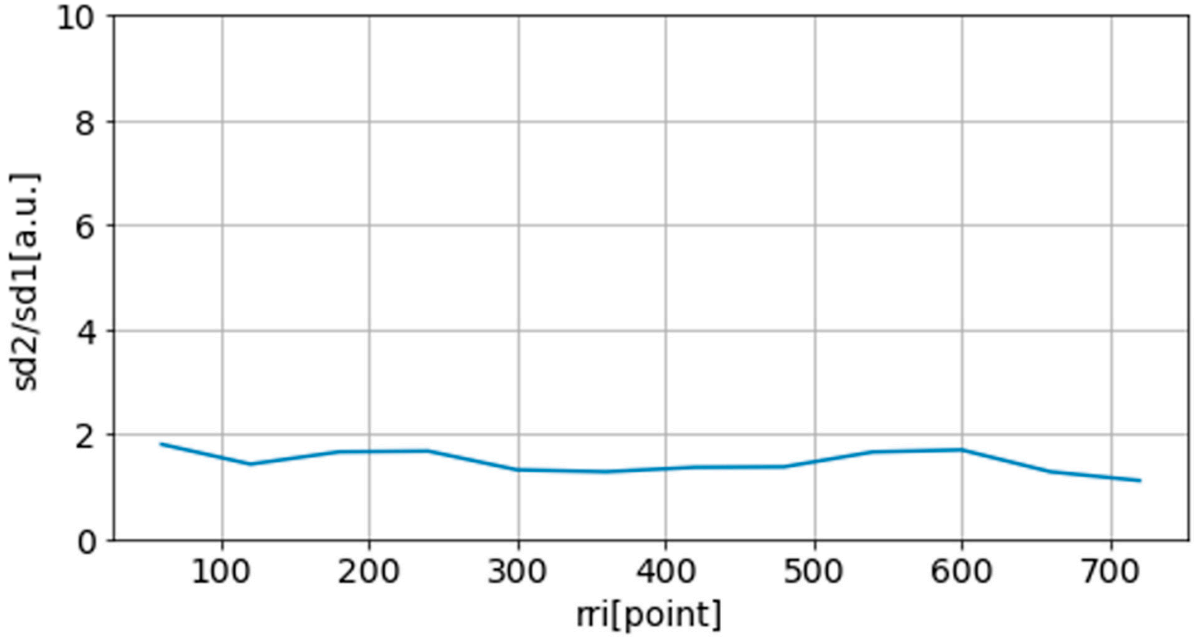


**Figure S10.** An example of *sd2/sd1* obtained from a stable measurement.

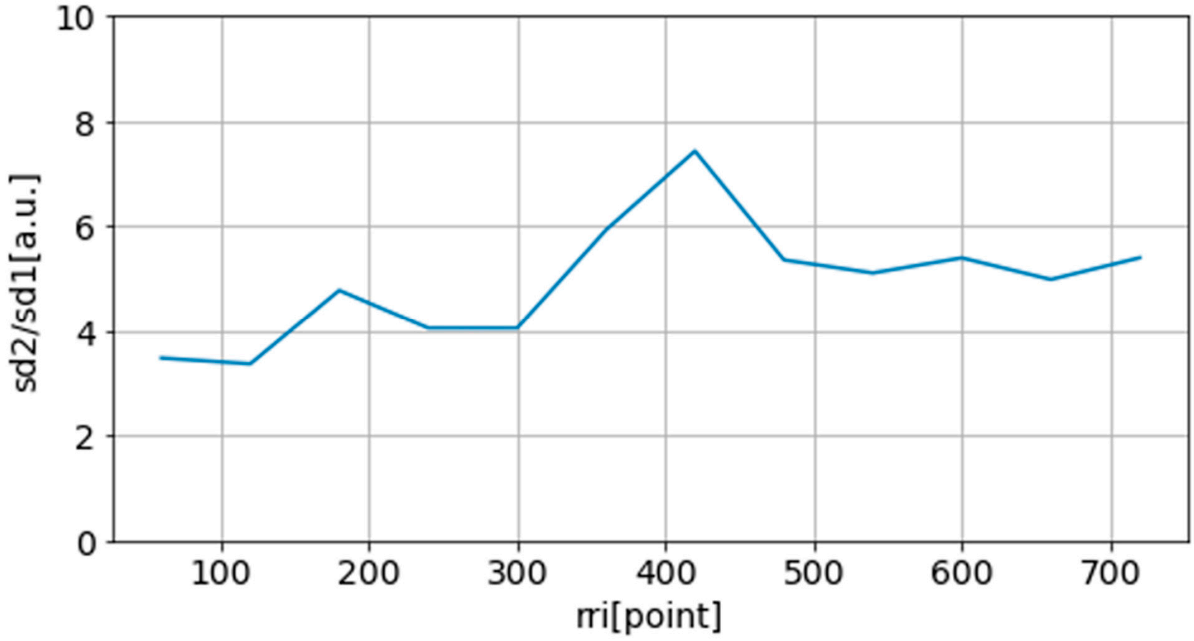


**Figure S11.** An example of *sd2/sd1* obtained from an unstable measurement.

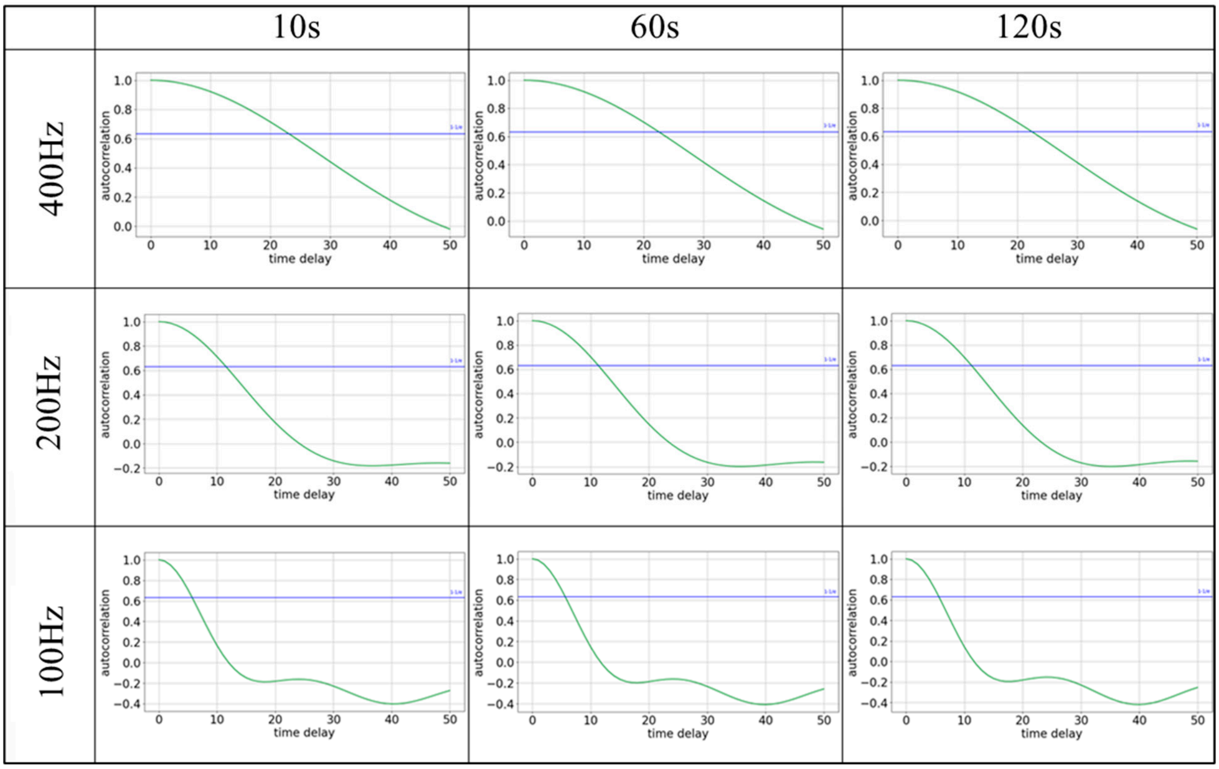


**Figure S12.** An example of the autocorrelation graph of the gPPG.

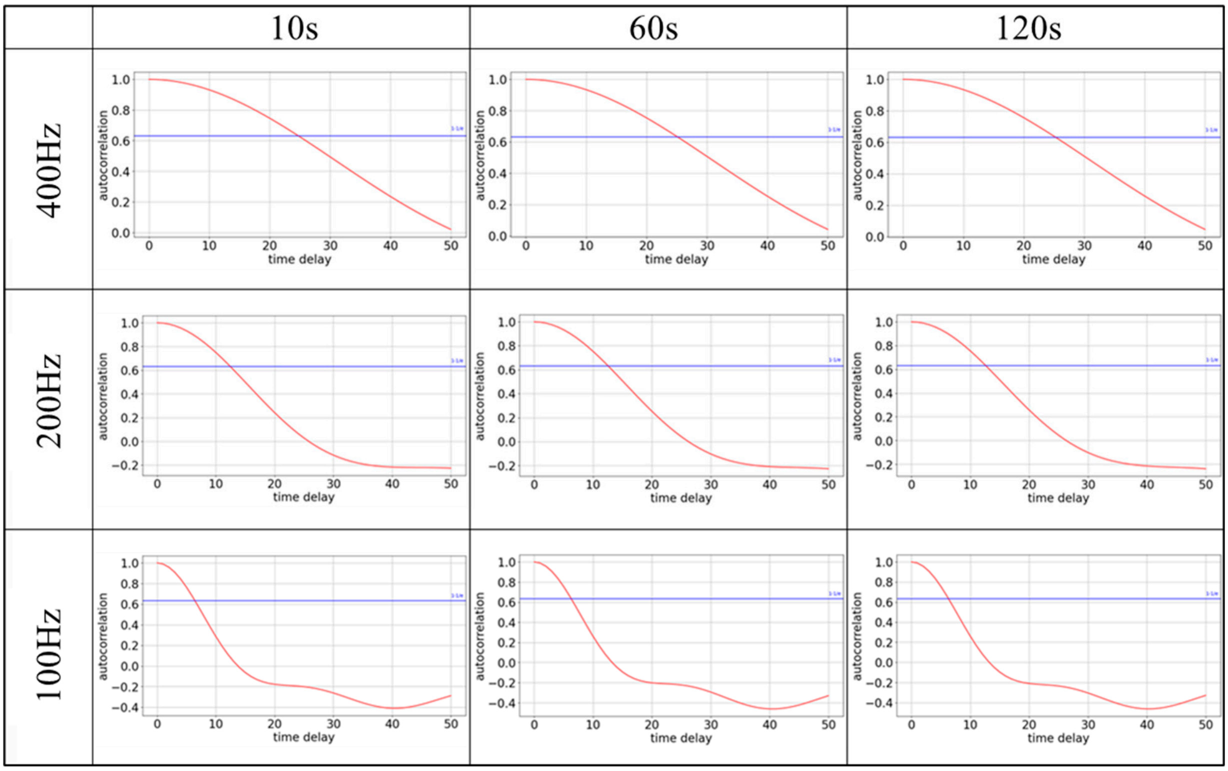


**Figure S13.** An example of the autocorrelation graph of the rPPG.

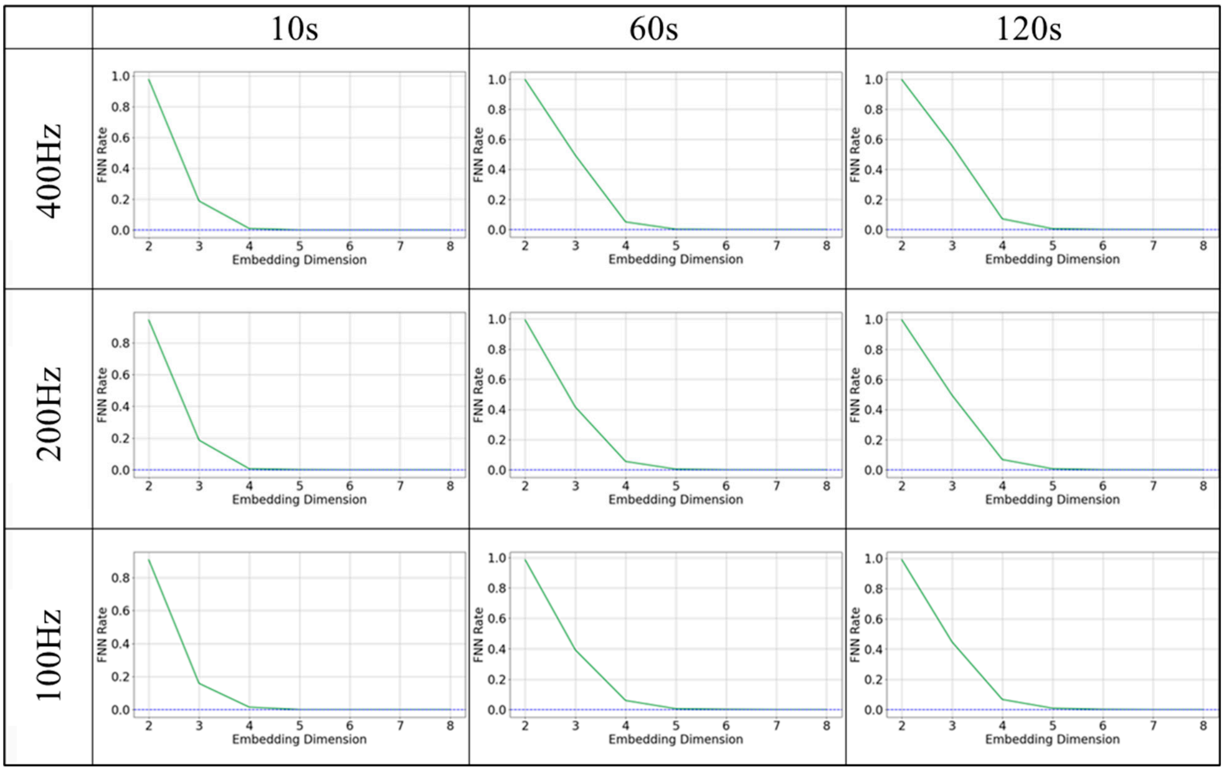


**Figure S14.** An example of dimension estimation by the false neighborhood method for gPPG.

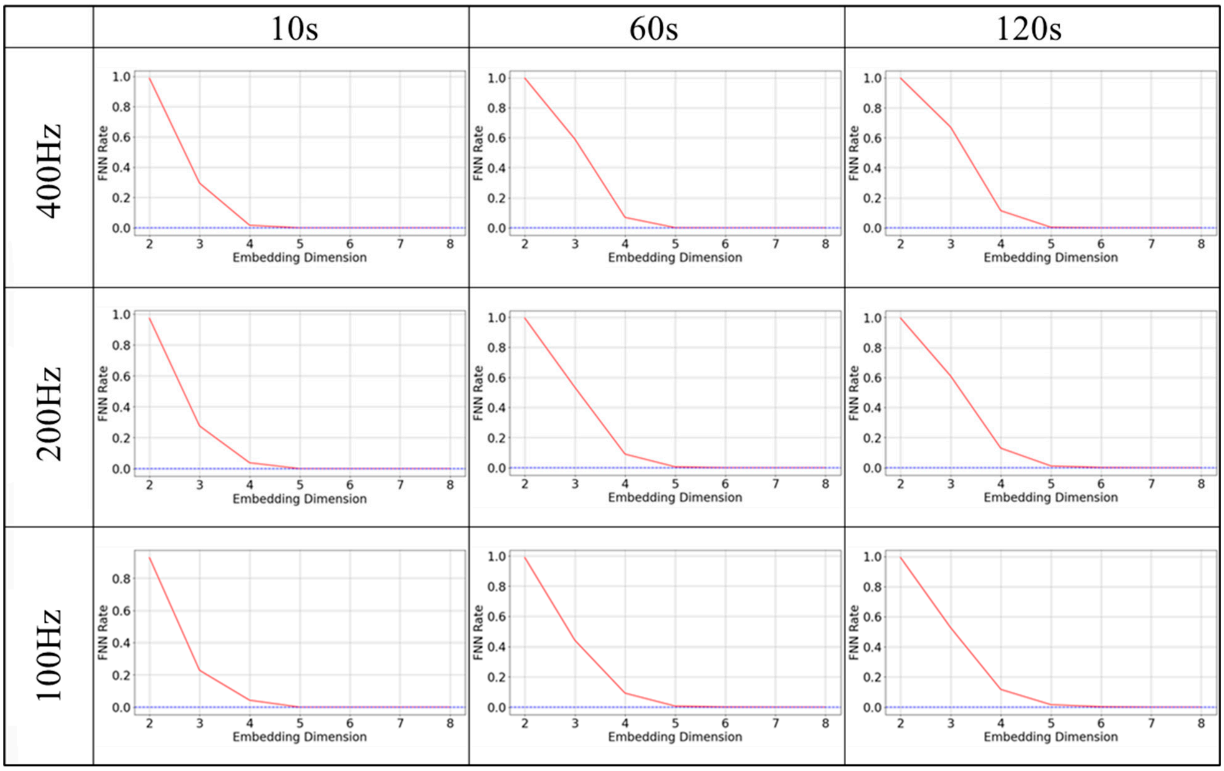


**Figure S15.** An example of dimension estimation by the false neighborhood method for rPPG.
